# Sex-dimorphic tumor growth is regulated by tumor microenvironmental and systemic signals

**DOI:** 10.1126/sciadv.ads4229

**Published:** 2024-12-06

**Authors:** Xianfeng Wang, Hongcun Bao, Yi-Chun Huang, Anindita Barua, Chun-Ming Lai, Jie Sun, Youfang Zhou, Fei Cong, Shangyu Gong, Chih-Hsuan Chang, Wu-Min Deng

**Affiliations:** Department of Biochemistry and Molecular Biology, Tulane University School of Medicine, Louisiana Cancer Research Center, New Orleans, LA 70112, USA.

## Abstract

Tumor growth and progression involve coordinated regulation by internal, microenvironmental, and systemic signals and often display conspicuous sexual dimorphism. The mechanisms governing the integration and coordination of these signals, along with their sex-based differences, remain largely unknown. Using a *Drosophila* tumor model originating from nonreproductive tissue, we show that female-biased tumor growth involves multifaceted communications among tumor cells, hemocytes, and neuroendocrine insulin-producing cells (IPCs). Notch-active tumor cells recruit hemocytes carrying the tumor necrosis factor–α (TNF-α) homolog Eiger to the tumor microenvironment (TME), activating the c-Jun N-terminal kinase (JNK) pathway in tumor cells, instigating the sexually dimorphic up-regulation of cytokine Unpaired 2 (Upd2). Upd2, in turn, exerts a distal influence by modulating the release of a *Drosophila* insulin-like peptide (Dilp2) from IPCs. Dilp2 then activates the insulin signaling in the tumor, thereby fostering sexual-dimorphic tumor growth. Together, these findings reveal a relay mechanism involving the TME and systemic signals that collectively control the sexual dimorphism of tumor growth.

## Teaser

Sex-dimorphic tumor growth involves multifaceted communication among tumor cells, immune cells, and neuroendocrine cells.

## INTRODUCTION

Continuous cell proliferation and growth represent a fundamental characteristic of tumor cells (*1*), and the tumor size/volume serves as a pivotal indicator of the tumor stage (*2*–*4*). However, the tumor growth rate exhibits considerable heterogeneity, varying among different cancer types and between sexes, and is often dependent on the interaction with the tumor microenvironment (TME), which comprises various components such as immune cells, cancer-associated fibroblasts (CAFs), and extracellular matrix (*5*, *6*). The regulation of tumor growth is linked to sustaining proliferative signaling, which can emanate from autocrine or paracrine interactions, from either neighboring tumor cells or nontumor cells in the TME. Both immune cells and CAFs in the TME have been documented as sources of such signals, including cytokines and chemokines, acting to sustain the proliferation of tumor cells (*7*, *8*).

Tumor growth is also regulated by systemic factors originating beyond the immediate vicinity of the TME, including hormones, cytokines, growth factors, inflammatory signals, and metabolites released by the endocrine, digestive, and immune systems (*9*). Insulin and insulin-like growth factor (IGF) are such signals that interact with the insulin-like receptor (InR), orchestrating integrated systemic regulation over metabolic and cell proliferation processes. Empirical evidence from animal and cell culture studies highlights the significance of the up-regulation of insulin and insulin-like growth factor signaling (IIS) activity during tumorigenesis (*10*). Disruption of IIS signaling has been shown to impede tumor growth in a transgenic mouse model of pancreatic neuroendocrine tumors (*11*). In a hyperinsulinemia mouse model, IIS accelerates breast cancer growth, and its blockade using inhibitors reduces tumor growth (*12*). In the same mouse model, a later study found that decreasing circulating insulin levels in these mice decreases tumor growth (*13*). Cell culture study using different cancer cells demonstrated that insulin accelerates cancer cell growth (*14*). Epidemiological studies in human populations also uncovered that hyperinsulinemia and increased IGF stimulate the growth of colon, liver, and pancreatic cancers by promoting cell proliferation and survival (*15*–*17*). This multifaceted interplay underscores the importance of understanding the nature of the signaling events between the tumor cells, the TME, and distant organs.

Sex differences in cancer incidence are well noted (*18*–*20*). Sex-biased tumor growth has been documented previously in various types of human cancers. For example, males typically present with larger renal cell carcinoma tumors than females at diagnosis, suggesting differences in tumor growth rates (*21*). In breast cancer, males tend to be diagnosed at a more advanced stage, indicating faster tumor growth compared to females (*22*). Similarly, men present with larger colorectal tumors at diagnosis. Multiple factors, including the immune system, sex hormones, sex chromosomes, and environmental exposures, may collectively contribute to these discrepancies (*18*, *20*, *23*–*25*). For example, sex-based differences in cancer-related signaling pathways such as c-Jun N-terminal kinase (JNK) and Janus kinase (JAK)–signal transducer and activator of transcription (STAT) signaling have been observed (*20*). JNK activity differs between females and males in acute liver injury and abdominal aortic aneurysm formation (*26*, *27*). The JAK/STAT pathway also shows sex-specific roles in medulloblastoma (*28*). Despite these advances, the precise mechanisms orchestrating sex-based differences in tumor growth, whether tumor cell autonomously or at the systemic level, remain largely unclear.

*Drosophila* has emerged as a potent model to decipher the mechanisms underlying tumorigenesis and the sex-linked nature of tumor malignancy (*29*), as well as for studying systemic regulation, particularly in the context of organ-organ communication crucial for cancer-induced wasting and cachexia phenotypes (*30*–*34*). In the *Drosophila* salivary gland imaginal ring (ImR) tumor model, continuous expression of a proliferative sustaining active Notch, the Notch intracellular domain (NICD), induces neoplastic transformation in a transition zone (TZ) region (*35*). The TZ cells are polyploid and enter error-prone polyploid mitosis upon Notch induction (*36*). Here, we show that the NICD-TZ tumors exhibit sexual dimorphism in tumor size, with female tumors significantly larger than male tumors. The tumor cells recruit circulating hemocytes to the TME, triggering JNK activation, which subsequently induces cytokine Unpaired 2 (Upd2)/leptin expression that promotes secretion of Dilp2 from insulin-producing cells (IPCs). Dilp2 then activates IIS signaling in the ImR tumor cells and regulates sexual dimorphism in tumor growth. All three pathways—JNK, Upd2, and IIS—have a female bias, which underlies the sexual dimorphism of tumor sizes. This relay of TME and systemic signals indicates that an intricate regulatory network reallocates the signals and resources needed to sustain tumor growth in a sexually dimorphic manner.

## RESULTS

### NICD-TZ tumors display sex-based differences in tumor size

**Fig. 1.**
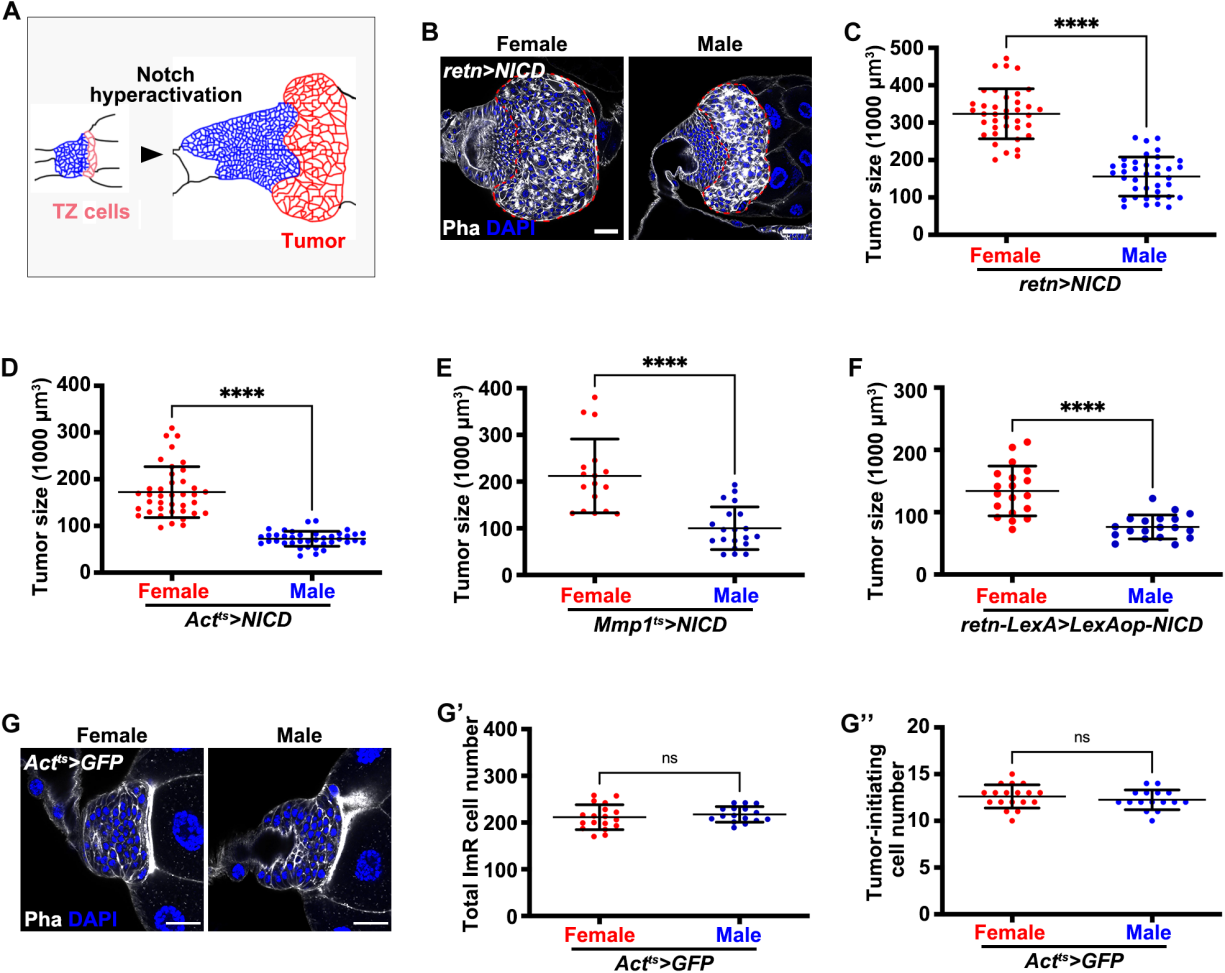
The NICD-TZ tumors exhibit sexual dimorphism in tumor size. (**A**) Graph depicting tumor formation in the *Drosophila* larval salivary gland ImR following Notch hyperactivation. (**B**) Larval salivary gland ImRs from female and male *Act^ts^>NICD* tumor stained with phalloidin (Pha; gray) and DAPI (blue). The tumor region is outlined with a red line. Scale bar, 20 μm. (**C** to **F**) Tumor volume of female and male *Act^ts^>NICD* tumors (7 days old) (C), *Mmp1^ts^>NICD* tumors (15 days old) (D), *retn>NICD* tumors (E), and *retn-LexA>LexAop-NICD* tumors (F). (**G**) Control salivary gland ImRs (*Act^ts^>GFP*) labeled with phalloidin (gray) and DAPI (blue). (**G′** and **G″**) Total cell counts in the salivary gland ImR (G′) and the posterior TZ (G″) of female and male controls (*Act^ts^>GFP*). Mean values and SD are shown. Student’s *t* test, ns, *P* > 0.05, ****P < 0.0001.

In a tumor model we developed in *Drosophila* larval salivary gland ImRs, continuous expression of the NICD, an active form of Notch (*37*), induces neoplastic transformation in the posterior TZ (*35*) (Fig. 1A; these tumors are referred as NICD-TZ tumors hereafter). When we measured the tumor volume following tumor induction with *retained* (*retn*)–*Gal4*, which shows Gal4 expression in the posterior half of the ImRs specifically (fig. S1), driving *UAS-NICD* expression, we observed a marked difference between female and male tumors (Fig. 1B). The average volume of female tumors is approximately 2.1 times that of the male tumors (Fig. 1C). The female bias in tumor size was reproduced when we used two other *Gal4* drivers, *Actin 5C* (*Act*)–*Gal4* and *matrix metalloproteinase 1* (*Mmp1*)–*Gal4* (fig. S1); the latter shows Gal4 expression in the TZ cells specifically in ImRs (Fig. 1, D and E, and fig. S1) (*35*, *36*). Moreover, applying the LexA/LexAop binary expression system to induce the NICD-TZ tumor by crossing *retn-LexA* with a newly generated *LexAop-NICD* transgenic line revealed that female tumors were consistently larger (~1.8-fold) than male tumors (Fig. 1F). The combination of the *LexA/LexAOP* and *Gal4/UAS* systems allows for the simultaneous induction of NICD-TZ tumors and manipulation of genes and pathways in other tissues and organs for studies of systemic regulation.

To rule out the possibility that salivary gland ImRs are sexually dimorphic in size during normal development, we measured their sizes at the third larval instar and found no noticeable difference between the two sexes (Fig. 1G). The average numbers of salivary gland ImR cells [male (M): 217.4 (*n* = 16); female (F): 211.5 (*n* = 18)] and the tumor-initiating TZ cells [M: 12.3 (*n* = 16); F: 12.6 (*n* = 18)] were also similar between the two sexes (Fig. 1, G′ and G″).

Next, we monitored the size of *Mmp1^ts^>NICD* tumors at various time points and found that female tumors were noticeably larger than male tumors at day 4 after tumor induction (ATI) at 29°C (fig. S2, A and A′). Analysis of tumor cell numbers showed that cells in female tumors increased more rapidly than in male tumors (fig. S2A″). As the NICD-TZ tumor cells are resistant to apoptosis (*36*), probably due to polyploidy and the expression of Diap1 (fig. S2B), we focused on whether the proliferation rate of tumor cells is different between the two sexes. Using the fly fluorescent ubiquitination-based cell cycle indicator (Fly-FUCCI) assay (*38*), we noticed a higher percentage of female tumor cells in G_2_ or M phase (F: 38.2%; M: 26.4%; fig. S2C). Analysis with the mitotic (M) phase marker phospho–histone H3 at Ser^10^ (pH3) further revealed the presence of more female tumor cells in M phase (fig. S2D; F: 8.97%; M: 4.26%). Additionally, male tumor cells exhibited larger nuclei and higher DNA content (fig. S2, E and F). These observations indicate a higher division rate of female tumor cells. Collectively, these findings suggest that the NICD-TZ tumors display tumor-specific sexual dimorphism in growth.

### JNK signaling exhibits sexual dimorphism in NICD-TZ tumors

**Fig. 2.**
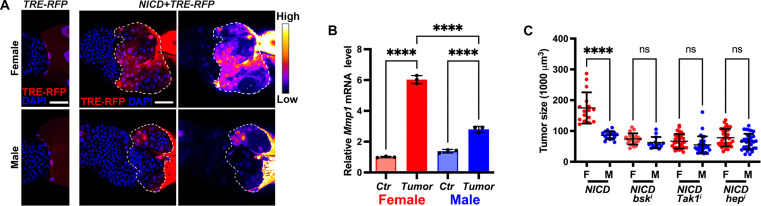
Up-regulation of JNK signaling in tumor cells and its role in tumor sexual dimorphism. (**A**) Salivary gland ImRs expressing JNK reporter TRE-RFP (red) were stained with DAPI (blue). (**B**) *Mmp1* expression in the control (Ctr) and tumor ImRs. (**C**) Tumor size analysis from the indicated genotypes. F, female; M, male. Tumor regions were contoured by dotted lines. Scale bars, 20 μm. Data represent mean ± SD, with statistical analysis conducted using one-way ANOVA Tukey’s multiple comparisons test. ns, *P* > 0.05, ****P < 0.0001.

Because of the crucial role of JNK signaling in NICD-TZ tumorigenesis (*35*), we measured the JNK transcriptional response element reporter *TRE-RFP* (*39*) to assess JNK activity in these tumors (Fig. 2A). Notably, TRE-RFP was detected in the majority of female tumor cells (70.1%, *n* = 14 tumors), but only in about half of the male tumor cells (49.7%, *n* = 14 tumors), and the fluorescent intensity of TRE-RFP appeared higher in female tumors (Fig. 2A). The level of JNK signaling was further analyzed by examining the expression of Mmp1, a known downstream target of the JNK pathway in *Drosophila* (*40*), which also exhibited higher up-regulation in female tumors (~6.0-fold increase in females versus ~2.0-fold in males) (Fig. 2B), indicating sexual dimorphism of JNK up-regulation in NICD-TZ tumors.

To determine whether differential up-regulation of JNK signaling contributes to the observed sexual disparity in tumor size, we manipulated JNK activity by silencing its components such as *basket* (*bsk*), *TGF-*β *activated kinase 1* (*Tak1*), and *hemipterous* (*hep*), the JNK kinase (JNKK) in *Drosophila* (*41*). Remarkably, knocking down *bsk* reduced the size of female tumors to approximately 42.3% (*n* = 21), and male tumors to approximately 72.2% (*n* = 13) (Fig. 2C). Tumor sizes were also reduced to 38.0% (*n* = 34) in female and 63.7% (*n* = 30) in male larvae, respectively, upon *Tak1* knockdown (Fig. 2C). Similar trend was observed when *hep* was knocked down (F: 44.6%, *n* = 34; M: 76.0%, *n* = 34) (Fig. 2C). In all three genetic backgrounds, the male and female tumor sizes appeared to be similar (Fig. 2C). Thus, reducing JNK signaling levels resulted in differential reduction in tumor sizes and mitigated their sexual dimorphism (Fig. 2C), suggesting the important role of JNK signaling in the sex difference of tumor growth.

### Hemocyte-produced Eiger activates JNK signaling in NICD-TZ tumors

**Fig. 3.**
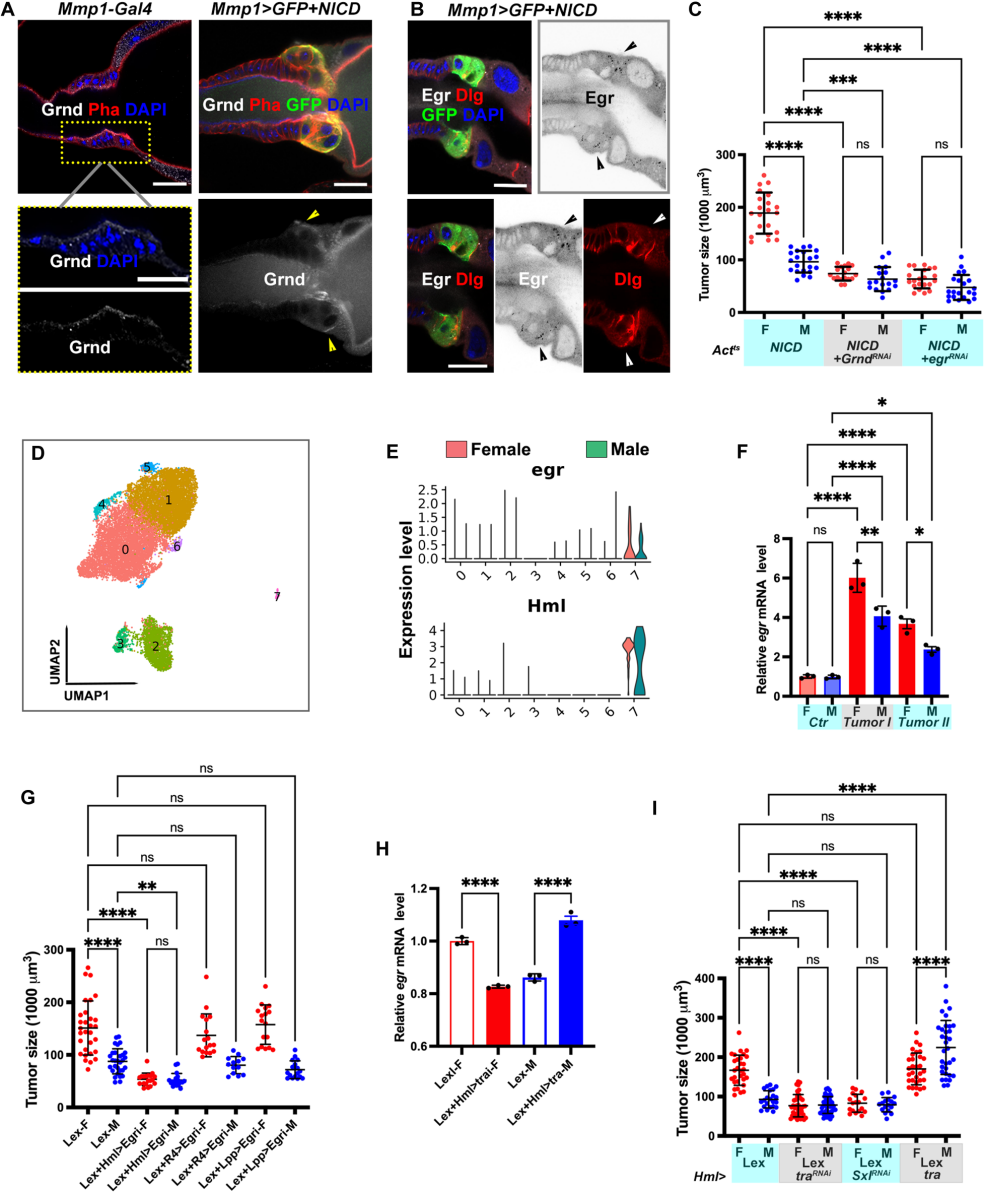
Ligand and receptor analysis of JNK signaling in NICD-TZ tumors. (**A**) Control (*Mmp1-Gal4*, male) and NICD-TZ tumor (*Mmp1>GFP+NICD*, male) ImRs stained with Grnd (gray), phalloidin (red), and DAPI (blue). Yellow arrowheads indicate basally localized Grnd in tumor cells. (**B**) Staining with Egr (gray), Dlg (red), and DAPI (blue) in NICD-TZ tumors (*Mmp1>GFP+NICD*, male). Black arrowheads indicate the presence of Egr in the tumor region, while white arrowheads indicate the mislocalization of Dlg. (**C**) Tumor size analysis from the indicated genotypes. (**D**) Uniform Manifold Approximation and Projection (UMAP) plot showing eight clusters of cells from scRNA-seq analysis of NICD-TZ tumor ImRs. (**E**) Violin plots showing coexpression of *egr* and the hemocyte marker *Hml* in cluster 7. (**F**) *egr* expression in hemocytes in the TME of *Act^ts^>NICD* (I) and *Mmp1^ts^>NICD* (II) tumors. (**G**) Tumor size analysis of *retn-LexA>LexAop-NICD* tumors (abbreviated as Lex tumors) with *egr* knockdown in either hemocytes (*Lex+Hml>egr^RNAi^*) or the fat body (*Lex+R4>Egr^RNAi^*, *Lex+Lpp>Egr^RNAi^*). (**H**) *egr* expression in the tumor-bearing hemocytes with *tra* knockdown or overexpression. (**I**) Tumor size analyses of Lex tumors and Lex tumors with *tra* or *Sxl* knockdown or *tra* overexpression in hemocytes. Scale bars, 20 μm. Data represent mean ± SD, with statistical analysis conducted using one-way ANOVA Tukey’s multiple comparisons test. ns, *P* > 0.05, *P < 0.05, **P < 0.01, ***P < 0.001, ****P < 0.0001.

The activation of JNK signaling can occur in either a ligand-dependent or ligand-independent manner (*42*). To distinguish these two possibilities, we used *Act^ts^-Gal4* to knock down the transmembrane receptors, Grindelwald (Grnd) (*43*) and Wengen (Wgn) (*44*), or the ligand Eiger (Egr) (*45*, *46*), a *Drosophila* homolog of tumor necrosis factor (TNF). Silencing *grnd* or *egr* using *Act^ts^-Gal4* resulted in a reduction in tumor size and diminished the sex difference of tumor size (Fig. 3C). Consistently, we induced NICD-TZ tumors in an *egr^1^* homozygous background and found that the tumor size was reduced (fig. S3A). In contrast, knockdown of *wgn* had no effect on tumor growth. These results suggest that the activation of JNK signaling in NICD-TZ tumors is ligand dependent and uses the Grnd branch to transduce the signal.

Epithelial cells normally prevent JNK activation during development by compartmentalizing the ligand and the receptor; Grnd is typically localized to the apical side of the epithelium, which faces the lumen in tubular structures (*43*). In NICD-TZ tumor cells, the disruption of apical-basal polarity, as indicated by mislocalization of an apical marker Patj (*47*, *48*) (fig. S3B), led to mislocalization of Grnd to the basal surface of tumor cells (Fig. 3A). The Egr ligand was also found to be associated with the tumor cells displaying compromised cell polarity, as indicated by the mislocalization of the basal-lateral marker Dlg (Fig. 3B). The female NICD-TZ tumor exhibited higher-level Egr signal when compared to the male tumor (fig. S3C).

Previous studies showed that Egr is produced in the fat body or hemocytes in *Drosophila* (*49*–*53*). To determine the source of Egr for JNK activation in the NICD-TZ tumor, we conducted a single-cell RNA-sequencing (scRNA-seq) analysis of ImR tumors (day 7 ATI at 29°C) and identified a distinct cell cluster enriched in *egr* expression (Fig. 3, D and E). These cells exhibited enrichment of *Hemolectin* (*Hml*), indicating their likely identity as hemocytes that may have been recruited to the TME. Consistent with the scRNA-seq results, labeling hemocytes with green fluorescent protein (GFP) (*Hml>GFP*) in larvae bearing *retn-lexA/lexAop-NICD* tumors revealed the attachment of GFP^+^ hemocytes to the tumor (fig. S3D), confirming the recruitment of hemocytes into the TME. Moreover, quantitative reverse transcription polymerase chain reaction (qRT-PCR) analysis of *egr* mRNA in circulating hemocytes from the hemolymph confirmed higher *egr* expression in female hemocytes (Fig. 3F). Consistently, Egr antibody staining of the hemolymph revealed that tumor-bearing female larvae showed higher levels of Egr associated with circulating hemocytes (fig. S3E).

To confirm that hemocytes are the source of the Egr signal activating JNK in the tumor, we specifically knocked down *egr* in hemocytes (*Hml-Gal4*) or the fat body (*R4-Gal4, Lpp-Gal4*) while inducing tumor formation with *retn-lexA*–driven *lexAop-NICD*. Depleting *Egr* in hemocytes led to a decrease in tumor size and a mitigation of tumor size sex difference, while *Egr* knockdown in the fat body with either *R4-Gal4* or *Lpp-Gal4* had no significant effect on tumor growth (Fig. 3G). In summary, our findings suggest that hemocytes are recruited to the TME and release Egr, in a female-biased manner, to activate JNK signaling in tumor cells to promote tumor growth.

### The sex determination cascade regulates the sex differences in TME hemocytes

The involvement of hemocytes in sex-dimorphic tumor growth prompted us to ask whether sex difference of hemocytes predetermines how they respond in the TME. To this end, we attempted to modify the sexual identity of hemocytes in tumor-bearing larvae by manipulating genes in the sex determination pathway. *Drosophila* sex determination depends on the X:A ratio, which leads to differential alternative splicing of *Sex lethal* (*Sxl*), the master regulator of sex in flies, and produces a functional protein in females, while the male form is inactive. Sxl controls the splicing of its own RNA and the downstream gene *transformer* (*tra*) (*54*). Manipulation of these genes can induce sex changes by altering the sex differentiation process. For example, loss-of-function mutations of *Sxl* or *tra* can cause genetically female flies (XX) to develop as males, while activation of *Sxl* or *tra* can induce female characteristics in genetically male flies (XY) (*55*). To determine if the sex of the hemocytes, which provide the source of JNK signaling, contributes to the sex dimorphism of tumor growth, we conducted qRT-PCR analysis of *egr* transcripts in the hemolymph of control and tumor-bearing larvae. We found that *egr* expression was down-regulated in female tumor–bearing larvae with hemocyte-specific *tra* knockdown. Conversely, *egr* expression was up-regulated in the hemolymph of male tumor–bearing larvae with hemocyte-specific *tra* overexpression (Fig. 3H). Consistently, hemocyte-specific knockdown of *tra* or *Sxl* resulted in a significant reduction of the tumor size in females but no change in males, while *tra* overexpression in the hemocyte increased tumor size in males but not in females (Fig. 3I). Additionally, the sex differences in tumor size were diminished when *Sxl* or *tra* was knocked down in hemocytes (Fig. 3I). These observations indicate that the sex identity of the hemocyte is crucial for the sex differences in tumor growth.

To determine if the sex identity of tumor cells intrinsically affects tumor growth, we manipulated *tra* in tumor cells using *retn-Gal4*. Knockdown of *tra* in female tumors resulted in a moderate reduction in tumor size, while in male tumors, it had no effect. Conversely, overexpression of *tra* did not alter the size of female tumors but caused a mild increase in male tumors. Despite these changes, the sex difference in tumor size persisted, albeit to a lesser extent (fig. S3F). Together, these results suggest that the sex identify of the tumor cells has a limited effect on the observed sex differences in tumor growth, and this effect is much smaller compared to the impact of *tra* manipulation in hemocytes.

### Tumor size sexual dimorphism arises from differential Upd2 up-regulation

**Fig. 4.**
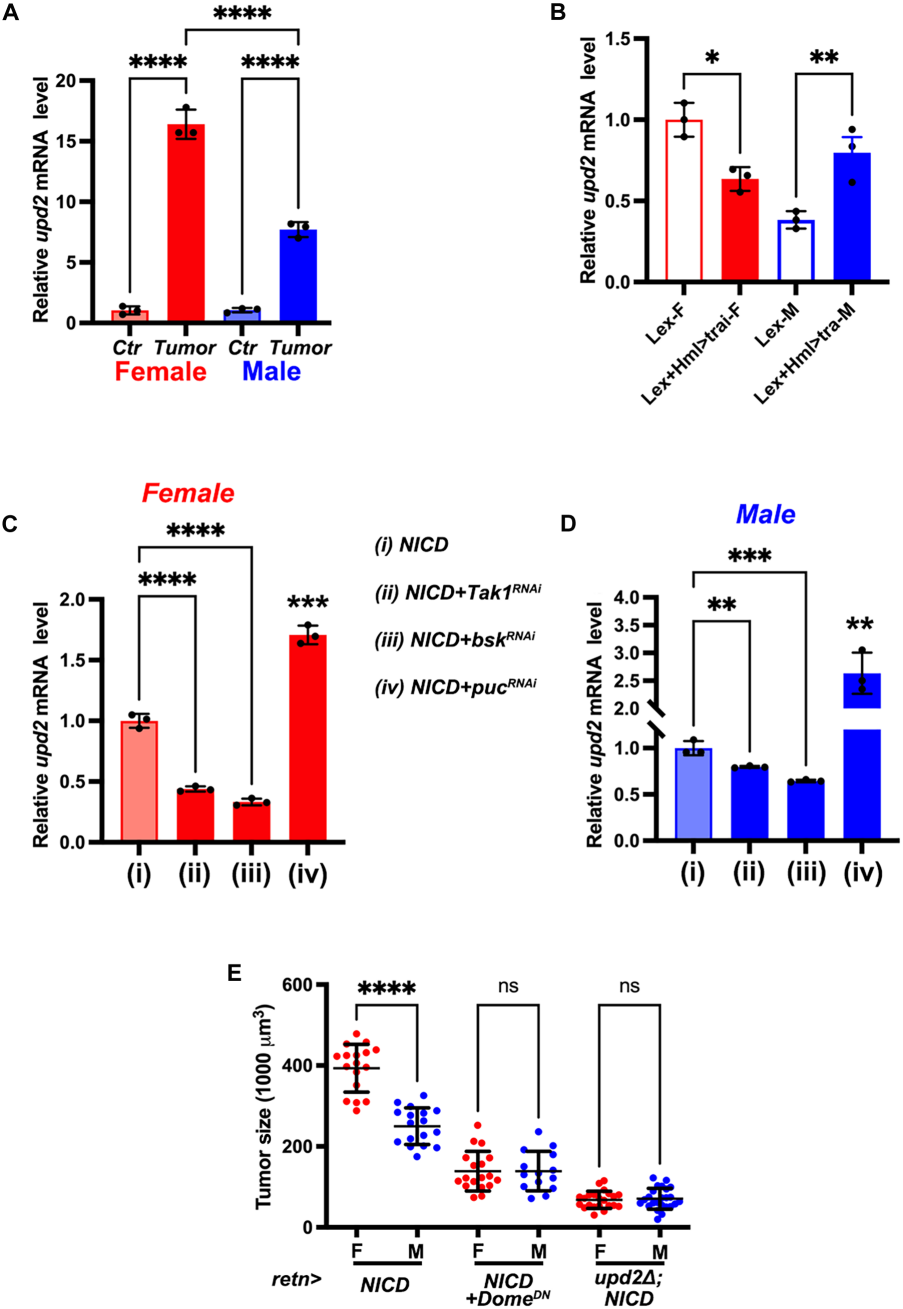
Upd2 mediates sexual dimorphism in tumor growth. (**A**) *upd2* mRNA levels in control (*Act^ts^>GFP*, Ctr) and NICD-TZ tumor (*Act^ts^>NICD*) ImRs. (**B**) *upd2* expression in the ImR tumors with *tra* knockdown or overexpression. (**C** and **D**) Expression of *upd2* in the indicated genotypes in female (B) and male (C) ImRs. (**E**) Tumor size analysis from *retn*-*NICD* tumors with overexpressing dominant negative form of Upd2 receptor Dome (*DomeDN*) or *upd2* deletion (*upd2*Δ). Data represent mean ± SD, with statistical analysis conducted using one-way ANOVA Tukey’s multiple comparisons test, except that data in (B) and (C) were analyzed with one-way ANOVA Dunnett’s multiple comparisons test. ns, *P* > 0.05, **P < 0.01, ***P < 0.001, ****P < 0.0001.

An important target of JNK signaling is the cytokine Upd2, a functional homolog of leptin and a ligand for the JAK/STAT pathway in *Drosophila* (*56*–*59*). qRT-PCR analyses revealed that *upd2* transcripts exhibited a more pronounced up-regulation in female tumors (Fig. 4A). Specifically, *upd2* expression was elevated 16.4 times in female tumors and 7.3 times in male tumors compared with the controls (Fig. 4A). Since we already showed that the sex-dimorphic up-regulation of *egr* expression in hemocytes is a source of tumor size sex difference, and *upd2* expression is potentially driven by JNK signaling, we examined the expression of *upd2* in tumors with sex-modified hemocytes. Consistently, knockdown of *tra* in hemocytes resulted in a decrease of *upd2* expression in the female tumors. Conversely, overexpressing *tra* in hemocytes resulted in a significant increase of *upd2* expression in male tumors (Fig. 4B).

To further confirm that up-regulated Upd2 results from increased JNK signaling in the tumor, we assessed the mRNA levels of *upd2* in NICD-TZ tumors with simultaneous knockdown of *Tak1*, or *bsk*, the *Drosophila* JNK, or the negative regulator *puc*. Comparison with sex-matched samples revealed that tumors with *Tak1* knockdown showed a 56.1% reduction of *upd2* transcripts in females and a 20.3% reduction in males. Similarly, tumor with *bsk* knockdown showed a reduction of *upd2* mRNA by 66.8% in females and 35.4% in males (Fig. 4, C and D). Conversely, tumors with knockdown of the JNK negative regulator *puc* exhibited an up-regulation of *upd2* expression (Fig. 4, C and D; female, 1.7 times; male, 2.6 times). Collectively, these findings indicate that *upd2* is up-regulated in the tumor in a sexually dimorphic manner, and the higher *upd2* levels in female tumors are induced by correspondingly higher levels of JNK signaling.

Next, we explored whether the sex difference in tumor size is attributed to the different levels of Upd2 up-regulation between male and female tumors. To validate Upd2 as a key factor responsible for tumor sexual dimorphism, we generated tumors in an *upd2* homozygous deletion mutant background (*upd2^Δ^*) (*60*). The *retn>NICD* tumors with *upd2* removal displayed a significant reduction in tumor size (F: reduced to ~17.3%; M: reduced to ~28.3%) and a reduced sex difference in tumor size (Fig. 4E). Similarly, knockdown of *upd2* in the tumor also resulted in reduced tumor size and diminished sex differences (fig. S4A), suggesting the crucial role of tumor-produced Upd2 in tumor size sexual dimorphism.

As Upd2 binds to the transmembrane cytokine receptor Domeless (Dome) to activate JAK/STAT signaling (*61*), we used a dominant negative form of Dome that lacks the intracellular domain (*UAS-Dome-DN*) (*62*) to suppress this pathway. Dome-DN, which can bind Upd2 but cannot activate JAK/STAT signaling, has been shown to sequester its ligands away from the endogenous Dome, thereby blocking pathway activation (*63*). Overexpression of Dome-DN also reduces the circulating level of Upd2, blocking both cell-autonomous and nonautonomous JAK/STAT signaling by sequestering ligands. Misexpression of *Dome-DN* in the *retn>NICD* tumor resulted in reduced tumor size (F: reduced to ~35.3%; M: reduced to ~55.5%). The sex difference in tumor size was diminished (Fig. 4E). This result contrasts with *Dome* knockdown in tumors, which had a mild effect on tumor size in both sexes and did not mitigate sex differences (fig. S4A). Since *Dome* knockdown only down-regulates JAK/STAT signaling cell autonomously, the marked effect of Dome-DN led us to conclude that the role of JAK/STAT signaling in tumor size sex dimorphism is likely due to tumor-released ligands that affect other tissues and organs.

### Tumor-produced Upd2 regulates insulin release from the IPCs

**Fig. 5.**
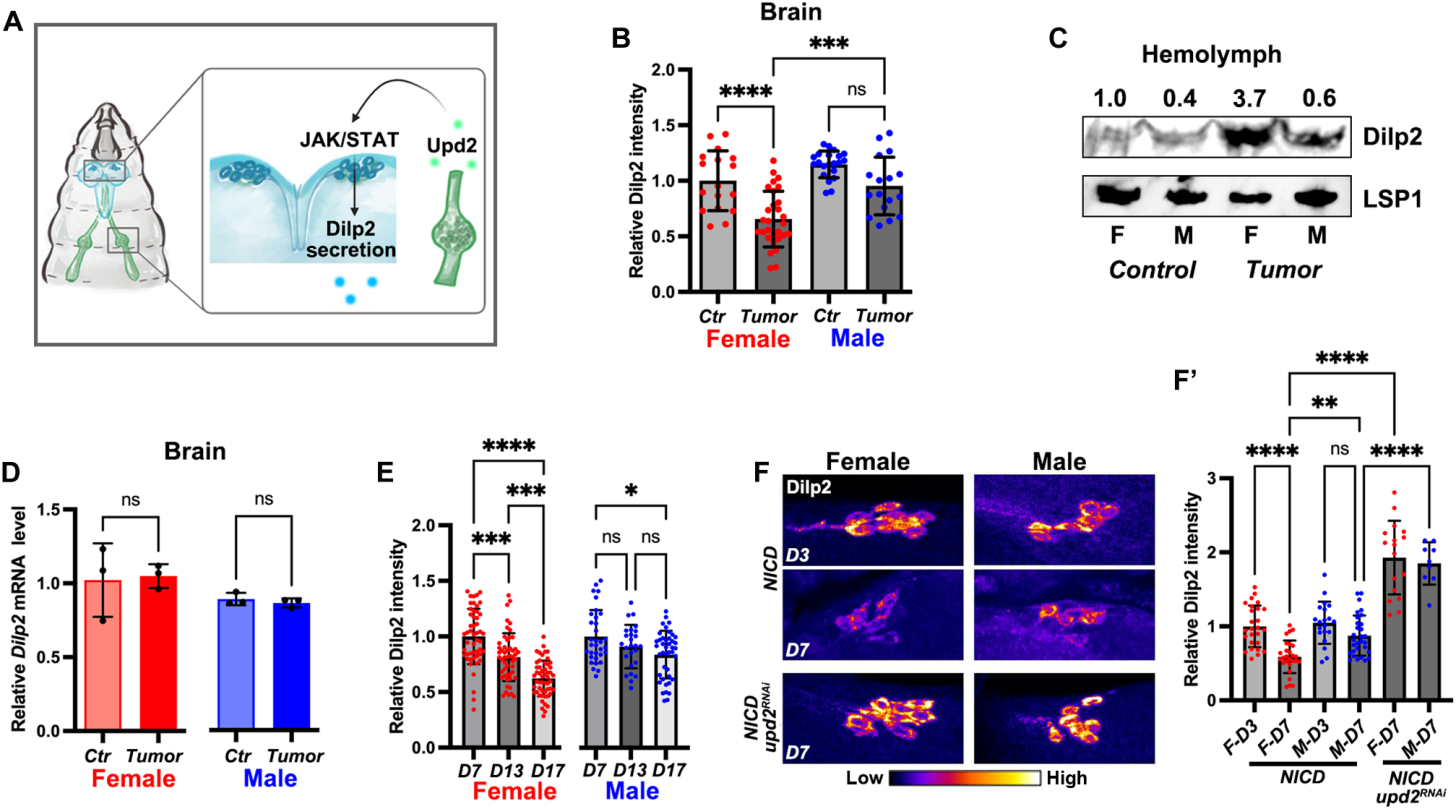
NICD-TZ tumor–produced Upd2 promotes Dilp2 secretion from IPCs. (**A**) A graph depicting the regulation of Dilp2 secretion is regulated by NICD-TZ tumor–produced Upd2. (**B**) Relative Dilp2 protein levels in control (*retn>GFP*, Ctr) and tumor (*retn>NICD*)–bearing larval brain IPCs. (**C**) Western blotting of Dilp2 in control and tumor-bearing larval hemolymph. Numbers indicate relative Dilp2 levels normalized to those of LSP1. (**D**) Dilp2 mRNA levels in control (Ctr) and tumor (*retn>NICD*)–bearing larval brains. (**E**) Dilp2 protein levels in female and male larval brain IPCs at different days after *Mmp1^ts^>NICD* tumor induction. (**F** and **F′**) Knockdown of *upd2* in NICD-TZ tumors decreases Dilp2 secretion in IPCs. (F) Dilp2 antibody staining of larval brains at different days ATI. (F′) Graph showing relative Dilp2 levels in day 3 and day 7 ATI, with or without *upd2* knockdown. Data represent mean ± SD, with statistical analysis conducted using one-way ANOVA Tukey’s multiple comparisons test. ns, *P* > 0.05, *P < 0.05, **P < 0.01, ***P < 0.001, ****P < 0.0001.

Upd2 is known for its role in the long-distance regulation of the secretion of *Drosophila* insulin-like peptide 2 (Dilp2), a peptide released by the IPCs in pars intercerebralis of the brain (*64*). Dilp2 binds to the InR in target cells, thereby activating the IIS signaling pathway, which promotes nutrient-dependent cell proliferation (Fig. 5A). On the basis of our observation of heightened Upd2 up-regulation in female tumors, we explored whether Dilp2 secretion from the IPCs is more pronounced in female tumor–bearing larvae. Consistent with findings from the adult fly brain (*64*), the larval brain also showed STAT reporter (STAT92E-GFP) expression in neurons adjacent to Dilp2-expressing IPCs (fig. S5A). Furthermore, the STAT reporter appeared much brighter in neurons adjacent to IPCs in tumor-bearing female larvae (fig. S5B). To test whether tumor-produced Upd2 can activate the JAK/STAT pathway in the brain, we knocked down *Dome* and *STAT92E* in the larval brain using *VGAT-Gal4*, which is expressed in GABAergic neurons and IPCs (fig. S4B) (*55*, *65*). We found that reducing JAK/STAT activity in the brain decreased tumor size and mitigated the sex difference in tumor size (fig. S4C). These findings suggest that tumor-produced Upd2 contributes to sex-specific differences in tumor growth by modulating JAK/STAT signaling in the brain.

As JAK/STAT signaling in the brain plays a role in Dilp2 releasing from IPCs in adult flies (*64*), we compared Dilp2 levels in IPCs between the control and the *retn>NICD* tumor-bearing larvae. The Dilp2 level was significantly lower in IPCs of female tumor–bearing larvae compared to the control or male tumor–bearing larvae (Fig. 5B). The decreased amount of intracellular Dilp2 in female tumor–bearing larval IPCs suggests increased Dilp2 secretion. We examined circulating Dilp2 in the larval hemolymph and found that Dilp2 levels were higher in the hemolymph of tumor-bearing larvae (Fig. 5C; female, 3.7 times; male, 1.5 times compared with sex-matched controls). Notably, brain *Dilp2* transcript levels did not differ between the two sexes (Fig. 5D). These results indicate that, rather than transcription, the key process causing the difference in Dilp2 levels between female and male larvae is Dilp2 release. Larvae derived from *Act^ts^>NICD* or *Mmp1^ts^>NICD* exhibited a developmental delay phenotype (*35*, *36*), providing an excellent model for assessing Dilp2 levels at different days ATI to track its release. In alignment with our findings in *retn>NICD*, *Mmp1^ts^>NICD* females exhibited a substantial reduction of Dilp2 levels in IPCs on day 13 and day 17 ATI when compared to day 7 ATI (Fig. 5E). These observations suggest that female IPCs released higher levels of Dilp2 into the hemolymph compared to male IPCs.

To validate that Dilp2 secretion is indeed controlled by Upd2, we conducted Dilp2 antibody staining on *Act^ts^>NICD* larval brains on day 3 and day 7 ATI. This analysis revealed a more pronounced reduction in Dilp2 levels in female IPCs than male counterparts between day 3 and day 7 ATI (Fig. 5, F and F′). Crucially, knocking down *upd2* in the tumor resulted in a significant retention of Dilp2 in IPCs in both sexes (Fig. 5, F and F′). Thus, the heightened up-regulation of Upd2 effectively governs the systemic regulation of Dilp2 secretion. These findings suggest that NICD-TZ tumors mimic the adipocyte by producing Upd2/leptin to regulate growth through insulin release.

### Differential IIS signaling drives sexual dimorphism in tumor growth

**Fig. 6.**
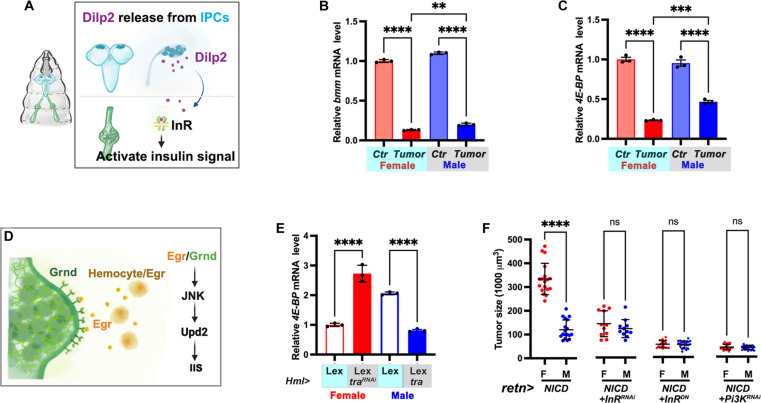
Differential IIS signaling in NICD-TZ tumors drives sex-dimorphic tumor growth. (**A**) Graph depicting the activation of IIS signaling in NICD-TZ tumors by IPC-released Dilp2. (**B** and **C**) Expression of *bmm* (B) and *4E-BP* (C) in control and tumor ImRs. (**D**) Graph illustrating the activation of IIS by Egr released from hemocytes. (**E**) Expression level of *4E-BP* in control tumors or tumors with *tra* knocking down or overexpression in the tumor-bearing hemocytes. (**F**) Graphs showing tumor sizes of indicated genotypes. Data represent mean ± SD, with statistical analysis conducted using one-way ANOVA Tukey’s multiple comparisons test. ns, *P* > 0.05, **P < 0.01, ***P < 0.001, ****P < 0.0001.

To assess IIS signaling activity in NICD-TZ tumors in both sexes (Fig. 6A), we used qRT-PCR to analyze the expression of two target genes, lipase *brummer* (*bmm*) and eukaryotic initiation factor 4E-binding protein (*4E-BP*), of Forkhead box subgroup O (FOXO), a transcription factor that negatively regulates the IIS pathway. A decrease of the transcription of *bmm* and *4E-BP* has been used to indicate up-regulation of IIS signaling in *Drosophila* tissues (*66*–*68*). The mRNA levels of both *bmm* and *4E-BP* were significantly reduced in NICD-TZ tumors. Female tumors showed a more pronounced reduction in their transcription. Specifically, *bmm* transcripts were reduced to approximately 13.1% in female tumors, and to about 20.0% in male tumors, while *4E-BP* transcripts were decreased to roughly 23.3% in female tumors and to approximately 46.7% in male tumors. These findings suggest higher up-regulation of IIS activity in female tumors than in male tumors (Fig. 6, B and C). To provide additional validation, we used a reporter containing GFP fused to the pleckstrin homology (PH) domain (GPH) to measure IIS activity. The tubulin-GPH (tGPH) reporter, using the *Drosophila* β-tubulin promoter, is ubiquitously expressed in the cell membrane, cytoplasm, and nucleus. The translocation of tGPH to the plasma membrane indicates an increase in IIS activity (*69*). Notably, female NICD-TZ tumor cells exhibited a higher GFP signal when tGPH was used (fig. S6, A, B, and B′). Furthermore, the assessment of IIS levels was conducted using phospho-Akt (pAkt-S505), an indicator of intracellular IIS activity. Consistent with expectations, pAkt levels were higher in the female ImRs based on antibody staining (fig. S6, C and C′) and Western blot analysis (fig. S6D).

To ascertain the potential impact of reduced IIS activity on tumor sizes and their sex differences, we conducted knockdown experiments targeting *phosphatidylinositol 3-kinase* (*Pi3K*) or *InR*, and also overexpressed a dominant negative form of *InR* (*InRDN*). Notably, the attenuation of IIS signaling resulted in a substantial reduction in tumor sizes (Fig. 6F). Significantly, this intervention ameliorated the sexual dimorphism in tumor size (Fig. 6F). Together, these findings suggest a crucial role of elevated IIS activity, induced by tumor-produced Upd2, in contributing to tumor growth and sex differences.

**Fig. 7.**
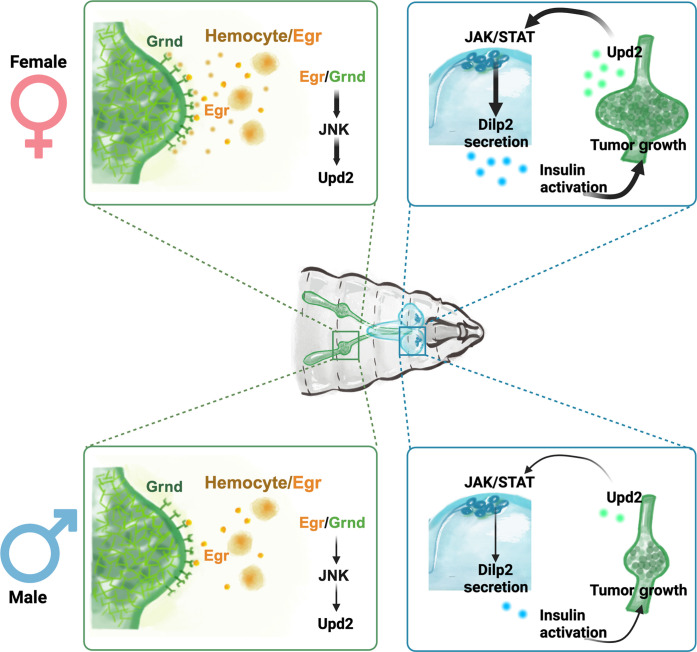
Schematic representation of the TME and systemic regulation underlying female-biased tumor growth. Hemocytes recruited to the NICD-TZ TME produce higher levels of TNF-α/Egr in female than in male larvae, triggering a cascade of female-biased events. These events include activation of JNK signaling in tumor cells, up-regulation of the critical JNK target leptin/Upd2, and remote activation of JAK/STAT signaling in GABAergic neurons. The process culminates in the release of Dilp2 from IPCs, leading to higher circulating levels of Dilp2, which activate IIS signaling in the tumor to promote female-biased growth.

Given the important role of hemocyte sex in the differential up-regulation of *egr* in tumor-bearing larvae, which in turn regulates IIS activity via tumor-released Upd2 (Fig. 6D), we examined IIS activity in tumors with hemocyte depletion of *tra* in female or hemocyte overexpression of *tra* in male. Knocking down *tra* in hemocytes resulted in higher level of *4E-BP* (indicating decreased IIS activity) in female tumors, and mis-expression of *tra* in male hemocytes caused lower level of *4E-BP* (indicating increased IIS activity) in male tumors, specifically (Fig. 6E). This finding confirms the regulatory relay involving hemocytes, tumor cells, and IPCs and highlights that the sex-dimorphic up-regulation of Egr in hemocytes is the root cause of the sex differences in NICD-TZ tumor growth regulation (Fig. 7).

## DISCUSSION

Our study reveals that tumor growth can be regulated by the TME and systemic factors in a sexually dimorphic manner. At the center of these regulations are the communications among the tumor cells, immune cells (hemocytes), and neuroendocrine IPCs (Fig. 7). Tumors recruit the hemocytes, which bear the TNF-α (Egr) signal, to the TME, resulting in activation of JNK signaling in tumor cells. An important target of JNK signaling in the tumor is cytokine Upd2/leptin, normally produced and released from the adipose tissue, the fat body, to induce the release of insulin-like peptides to regulate systemic growth. Remarkably, these tumor cells mimic adipocytes by sending the Upd2/leptin signal to regulate insulin release, thereby controlling tumor growth. Female larvae, in particular, display a higher degree of Egr up-regulation, triggering the downstream cascade of events that favor the faster growth of the female tumor. The Egr/JNK, Upd2-JAK/STAT, and IIS pathways have emerged as critical players in both the TME and systemic orchestration of tumor growth and contributing to the observed sexual dimorphism.

The activation of JNK in NICD-TZ tumor cells involves its ligand Egr and is dependent on the Grnd receptor, which is normally localized on the apical side of epithelial cells facing the lumen. NICD-TZ tumor cells exhibit compromised cell polarity, with Grnd present at the exterior surface to facilitate interaction with Egr. The interaction between Egr and Grnd in cells with polarity defects aligns with previous findings related to cell elimination (*53*), but the source of Egr appears to be different. In *Drosophila*, hemocytes and the fat body are two major Egr-producing tissues. The study on cell competition and elimination showed that the fat body is the source of Egr, binding to polarity-defective cells where Grnd is mislocalized (*53*). Fat body–derived Egr also serves as an adipokine to mediate nutrient response through the inhibition of Dilp secretion (*50*), and is essential for robust humoral immune response to microbes (*70*). Hemocytes, on the other hand, have been identified as the source of Egr in imaginal disc tumors, resulting from disruption of cell polarity genes (*49*, *71*), as well as in apoptosis-induced proliferation in wing and eye discs (*52*). In both scenarios, hemocytes appear to be recruited to the vicinity of the imaginal discs. This parallels the situation in our NICD-TZ tumors generated in salivary gland ImRs, where hemocytes are recruited to the TME. Thus, it is likely that Egr produced by the fat body is secreted into the hemolymph and functions in endocrine (long-range) signaling, whereas hemocyte-produced Egr may primarily participate in paracrine (short-range) signaling. TNF-α has been shown to be involved in both endocrine and paracrine signaling in ovarian cancer (*72*). Further studies are necessary to determine whether there are differences in the sequence or modification of Egr that enable it to function as either a paracrine or endocrine signal.

The fat body is also the source of Upd2, the functional leptin ortholog, which controls growth remotely through IIS signaling during development (*64*, *73*, *74*). Upd2 regulates the release of Dilps in response to alterations in nutrient status (*64*, *73*). The up-regulation of Upd2 in NICD-TZ tumors appears to involve molecular mimicry of the fat body by promoting the secretion of Dilp2. The heightened Upd2 levels observed in female tumors result in an augmented release of Dilp2, consequently accelerating tumor growth. The target tissue of tumor-derived Upd2 appears to be neuronal cells adjacent to the IPCs (fig. S5). Although this finding is during the larval stage, the target cell is similar to fat body–derived Upd2 observed previously in adult flies (*64*). Our study thus suggests that tumor-produced Upd2 targets IPCs in the brain to regulate tumor growth. Additionally, Upd2 has been shown to target other tissues such as the blood-brain barrier, intestine, fat body, hemocytes, muscles, and the ring gland (*56*, *75*–*82*), although their impact on the NICD-TZ tumors is not yet clear. Although the structural distinctions between Upd2 and human leptin are evident, it is noteworthy that human leptin has been implicated in diverse human cancer types (*83*, *84*), with elevated leptin levels linked to the aggressiveness and prognosis of breast cancer (*85*, *86*). Furthermore, higher circulating levels of leptin in prostate cancer patients were found to be associated with an increased risk of larger-volume tumors (*87*). These correlations suggest that the link between Upd2/leptin and tumor size is likely conserved.

In another *Drosophila* tumor model, brain tumors driven by the *lethal(3) malignant brain tumor* [*l(3)mbt*] mutation exhibit male-biased malignancy due to male-specific up-regulation of the chromatin reader, PHD finger protein 7 (Phf7) (*29*). This mechanism appears distinct from that observed in NICD-TZ tumors, where Phf7 does not show clear sex-specific difference between male and female tumors. The root cause of the sex dimorphism of the NICD-TZ tumor size appears to be the sex difference in hemocytes. Their response to tumors may determine the growth rate of this tumor model in different sexes. The faster growth of female tumors caused by higher Egr/JNK signaling can be mitigated by modulating sex determination genes in the hemocyte. This is consistent with previous studies that demonstrated that adult flies display sex-dimorphic innate immunity in response to infections (*88*), and Toll pathway activity exhibits sexual dimorphism after bacterial infection (*89*). As previous studies are performed in adult flies, our study suggests that larval hemocytes can have sex-dimorphic response to tumor cells. It is yet unclear why female hemocytes in the TME show higher levels of the Egr signal. The TME may have recruited more hemocytes in females, but this is not likely, as we did not detect substantially different numbers of hemocytes in the female TME compared to the male. Alternatively, the sex determination pathway may have an impact on the expression level of Egr in hemocytes upon tumor induction, warranting further investigation. Nonetheless, the pivotal role played by the sex determination cascade in tumor growth sex dimorphism suggests that genetic differences between the two sexes precede the hormonal and metabolic differences in the regulation of tumor growth, a principle likely shared in mammalian tumors.

## MATERIALS AND METHODS

### Fly stocks and genetics

Flies were raised on Bloomington Drosophila Stock Center (BDSC) cornmeal food. To eliminate the possibility that observed phenotypes were due to the fly genetic background, a 10-generation back-cross was conducted for the UAS-NICD line. The following Gal4 drivers were used in this study to induce ImR tumors: *Act-Gal4*, *Mmp1-Gal4*, and *retn-Gal4*. *Tub-Gal80ts* was used to control Gal4 activity. *UAS-egrRNAi* (VDRC#104538) and *UAS-grndRNAi* (VDRC#104538) were gifts from D. Bilder. Detailed genotypes were listed in table S2. Gonad size served as the primary criterion for sex determination. In some experiments, red fluorescent protein (RFP) on the X chromosome was used to differentiate female and male larvae. Male flies carrying UAS-RFP on the X chromosome were crossed with virgin females of the appropriate genotype. In the progeny, females were RFP positive, while males were RFP negative.

### Tumor size and cell number analysis

For tumor size measurement experiments, 20 virgin females (2 to 3 days old) were cross with 15 males of the appropriate genotype. Adult flies were transferred every 2 days for egg collection. Eggs from control tumors and tumors with gene knockdown or overexpression were collected and raised in the same condition. Tumors from the same experimental group were dissected at the same day for tumor size analysis. Tumor size analysis was performed as previously described (*36*). Cell numbers were manually counted using ImageJ (Fiji) software with the “multi-point” function (*90*).

### Real-time qRT-PCR

Total RNA was extracted from larval tissues using the RNAprep kit (Zymo Research; R2070) following the provided protocol. cDNA synthesis was performed with the Reverse Transcription Kit according to the manufacturer’s instructions (Invitrogen; 2508657). qPCR analysis was carried out as previously described (*91*). The reaction mixture containing cDNA, relevant primers, and iTaq Universal SYBR Green Supermix (Bio-Rad, #172-5124) were loaded onto a 96-well plate. The CFX96 Touch Real-Time PCR System (Bio-Rad) was used for qPCR analysis. Primer sequences used for qPCR are provided in table S1. Glyceraldehyde-3-phosphate dehydrogenase (GAPDH) mRNA was used for normalization.

### Immunohistochemistry and confocal imaging

Samples were dissected, fixed, and stained as previously described (*92*). The following primary antibodies were used in this study: rat anti-Dilp2 (a gift from Dr. P. Léopold, 1:200), mouse anti-Grnd (a gift from D. Bilder, 1:500), mouse anti-Patj (a gift from Y. Hong, 1:1000), mouse anti–β-galactosidase (Promega, 1: 200), mouse anti-Dg [(*93*), 1:1000], rabbit anti-Egr (a gift from Dr. M. Miura, 1:50), rabbit anti-pAkt (Ser^505^) (Cell Signaling, 1:100), and rabbit anti-pH3 (Millipore, 1:200). Secondary antibodies conjugated with Alexa Fluor 488, 546, or 633 (Invitrogen, 1:400) were incubated at room temperature for 2 hours. DAPI (4′,6-diamidino-2-phenylindole) or Hoechst was used to stain DNA. Alexa Fluor 488–, 546–, or 633–conjugated phalloidin was used to label F-actin (1:50, Invitrogen). Samples were mounted and imaged using a Zeiss LSM 800 or Zeiss LSM 980 confocal microscope.

### Western blot

Western blotting was performed as described previously (*94*). Hemolymph protein extracts were obtained from 150 larvae for each sex as described (*95*). Control and tumor ImR protein extracts were obtained from 200 larvae for each sex. Antibodies were used at the following dilutions: anti-Dilp2 [(*95*), a gift from Z. Gong, Zhejiang University, and Y. Li, Chinese Academy of Sciences, China, 1:2000], anti-LSP1 (Cell Signaling, 1:1000), anti-pAkt (Cell Signaling, 1:1000), and anti-actin (Developmental Studies Hybridoma Bank, 1:500). Goat antimouse or antirabbit horseradish peroxidase (HRP)–conjugated secondary antibody (Invitrogen, 1:10,000) were used to detect primary antibodies. SuperSignal West Atto Ultimate Sensitivity Chemiluminescent Substrate (Thermo Fisher Scientific, A38555) was used for detection.

### Fluorescence intensity analysis

Images were acquired with the Zeiss LSM 800 confocal microscope using the same laser power and gain value settings. Mean fluorescence intensity from each section was quantified using ImageJ (Fiji) software (*90*).

### scRNA-seq library preparation sequencing and data analysis

Cell suspensions were obtained by adapting previously established protocols (*96*, *97*). Briefly, ImR tumors were dissected and dissociated with papain. Viability and cell concentration were assayed using a hemocytometer (Hausser Scientific). Single-cell libraries were generated using the Single Cell Bead Kit Cell 3′ Library & Gel Bead Kit v2 following the 10X Genomics protocol. The libraries were sequenced using the NovaSeq 6000 (Illumina) instrument. Data analysis was performed on the Cell Ranger output using Seurat (version 4.0) (*98*).

### Quantification and statistical analysis

Data analysis was conducted using GraphPad Prism software. Unpaired *t* tests were used for two-sample comparisons, and one-way analysis of variance (ANOVA) with Dunnett’s or Tukey’s multiple comparisons test was used for multiple-sample comparisons. Specific statistical approaches for each figure are indicated in the figure legends.

## Supplementary Materials

### This PDF file includes:

Figs. S1 to S6 Tables S1 and S2 References
