# Supplementary material for: Sex-dimorphic tumor growth is regulated by tumor microenvironmental and systemic signals
Source: Sci Adv. 2024 Dec 6;10(49):eads4229. doi: 10.1126/sciadv.ads4229 (PMC11623276; doi:10.1126/sciadv.ads4229)
Supplement: Supplementary file 1 — Figs. S1 to S6 Tables S1 and S2 References [file sciadv.ads4229_sm.pdf]

Science Advances
AAAS

Supplementary Materials for

Sex-dimorphic tumor growth is regulated by tumor microenvironmental and systemic signals

Xianfeng Wang *et al.*

Corresponding author: Wu-Min Deng, wdeng7@tulane.edu

Sci. Adv. **10**, eads4229 (2024)
DOI: 10.1126/sciadv.ads4229

This PDF file includes:

Figs. S1 to S6
Tables S1 and S2
References

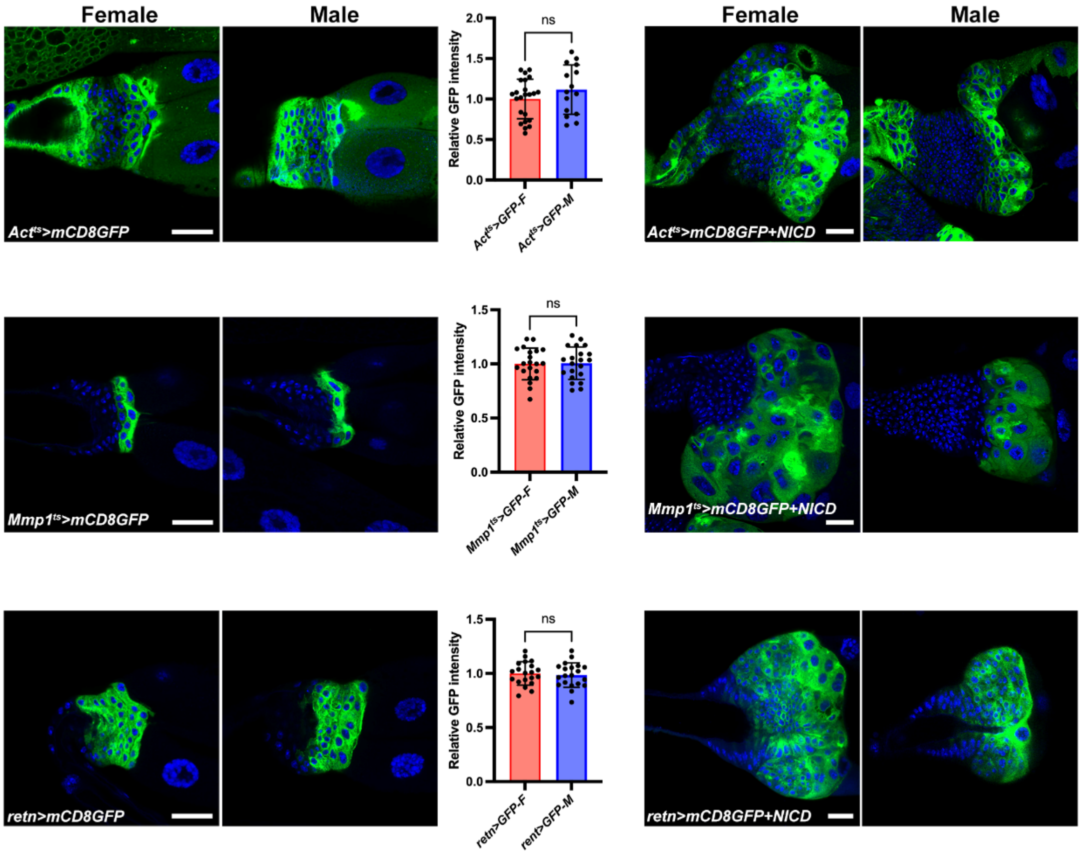

Fig. S1. NICD-TZ tumors induced by different Gal4 drivers. Left panel: GFP indicates the expression pattern of Gal4s in larval salivary gland ImRs. Right panel: Tumors can be induced in the posterior transition zone of the ImR upon NICD induction using different Gal4 drivers. Scale bars: 20 µm. Graphs showing quantification of GFP intensity in the TZ cells from the indicated genotypes.

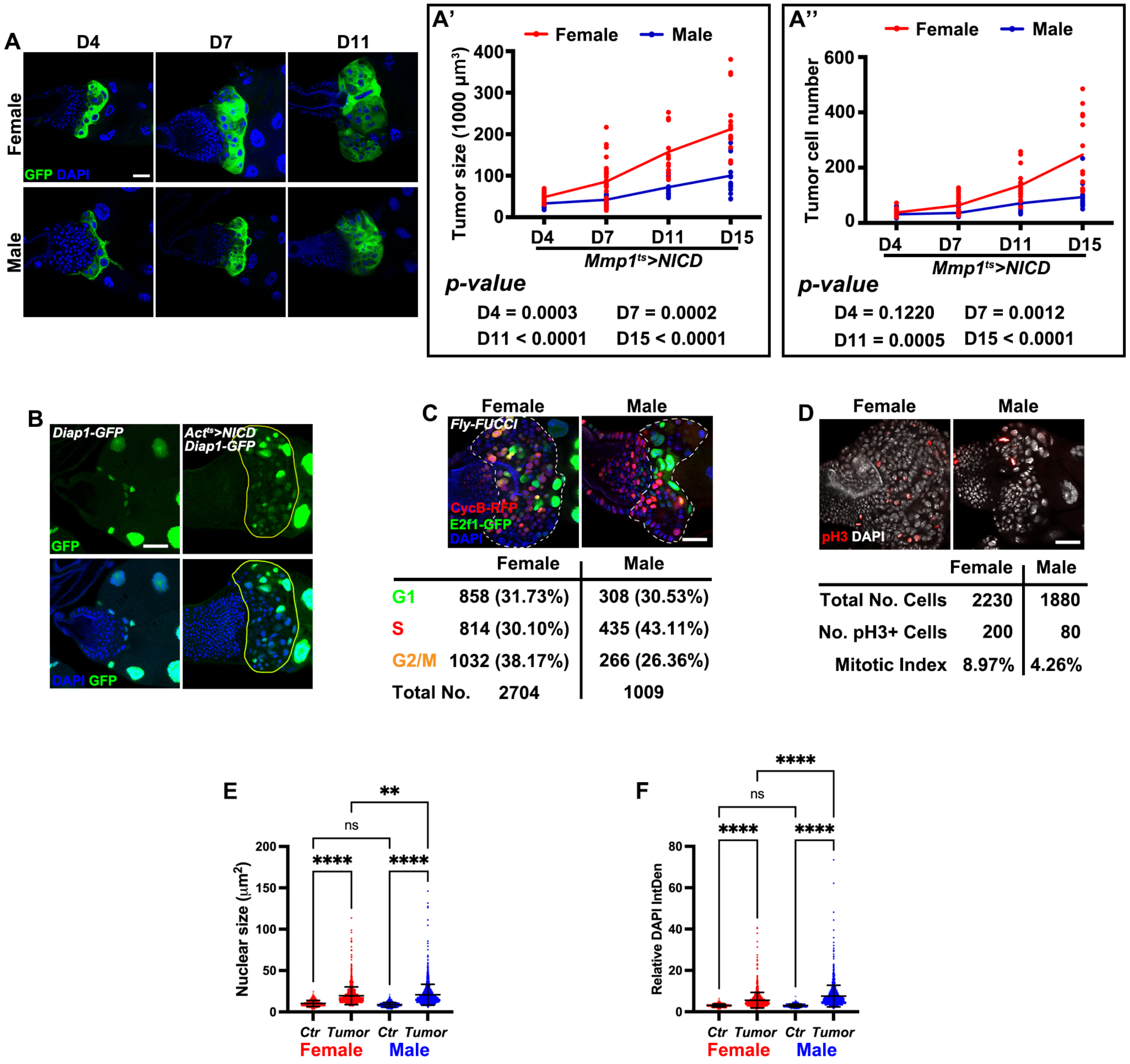

	Female	Male
G1	858 (31.73%)	308 (30.53%)
S	814 (30.10%)	435 (43.11%)
G2/M	1032 (38.17%)	266 (26.36%)
Total No.	2704	1009

	Female	Male
Total No. Cells	2230	1880
No. pH3+ Cells	200	80
Mitotic Index	8.97%	4.26%

Fig. S2. Growth analysis of NICD-TZ tumors. (A) Images show tumor growth of *Mmp1^{ts}>NICD* at different day points. Tumor volumes (A') and cell numbers (A'') were analyzed for female and male tumors at different days. P-values present unpaired two-tailed t test between female and male samples on the same day. (B) Diap1-GFP expression in control and tumor ImRs. The tumor region is outlined by yellow lines. (C) Female and male salivary gland ImRs with fly-FUCCI expression stained with DAPI (blue). The tumor region is marked by white dashed lines. The table indicates the total number of G1, S, G2/M tumor cells observed. (D) Female or male *Act^{ts}>NICD* ImR tumors were stained with pH3 antibody (red). The table indicated the total number of pH3 positive cells number, and the total tumor cell number observed. (E, F) Nuclear area and relative DAPI integrated density (IntDen) were analyzed for cells from the anterior non-tumor region (serving as the internal control, Ctr) and the posterior tumor cells. Due to variations in DAPI fluorescence across different scanning sections and experimental rounds, we normalized the tumor cell IntDen using the anterior-region cells, which are diploid, as a reference for each image. Scale bars, 20 μm.

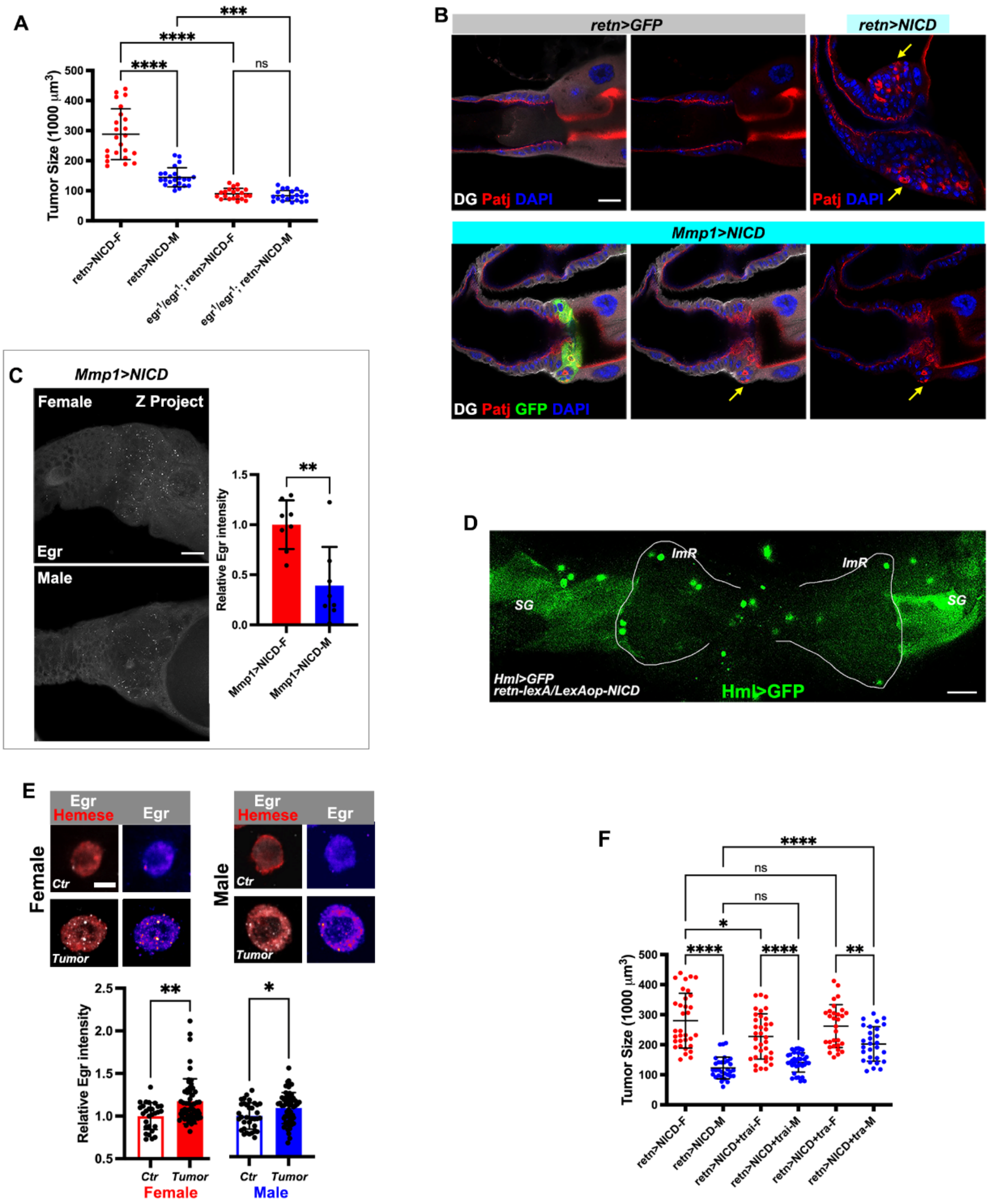

Fig. S3. Analysis of the impact tumor microenvironment and intrinsic sex difference on tumor growth. (A) Quantification of tumor size of *retn>NICD* and *egr*^{1}*/egr*^{1}*; retn>mCD8GFP/UAS-NICD* tumors. (B) Staining of control and NICD-TZ tumor ImRs with antibodies against apical-basal polarity markers Dystroglycan (Dg, white) and Patj (red), respectively. Yellow arrows indicate mislocalization of the apical maker Patj. Scale bars, 20 μm. (C) Z projection of female and male *Mmp1>NICD* ImRs with anti-Egr antibody staining, showing a stronger signal in the female tumor (top panel). Scale bars, 20 μm. The graph shows quantification of relative Egr mean intensity. Egr intensity was quantified on the surface of samples in both tumor and non-tumor regions from individual images. The intensity from the non-tumor region was subtracted from that of the tumor region to determine the Egr intensity for each sample. The 'relative' refers

to the comparison with the Egr intensity from female tumors. (D) A snapshot of live-imaging samples showing the attachment of hemocytes (labelled with Hml>GFP) around the tumor region (outlined by white lines). Scale bars, 20 µm. (E) Hemocytes from control and tumor-bearing larvae were stained with the hemocyte marker Hemese (red) and Egr (white) antibodies. The graph shows the relative intensity of Egr. Scale bars, 5 µm. (F) Tumor size analysis of the indicated genotypes. Data represent mean ± SD, with statistical analysis conducted using one-way ANOVA with Tukey's multiple comparisons test, except Student's t test was used for C. ns, $p > 0.05$; $*p<0.05$, $**p<0.01$, $****p < 0.0001$.

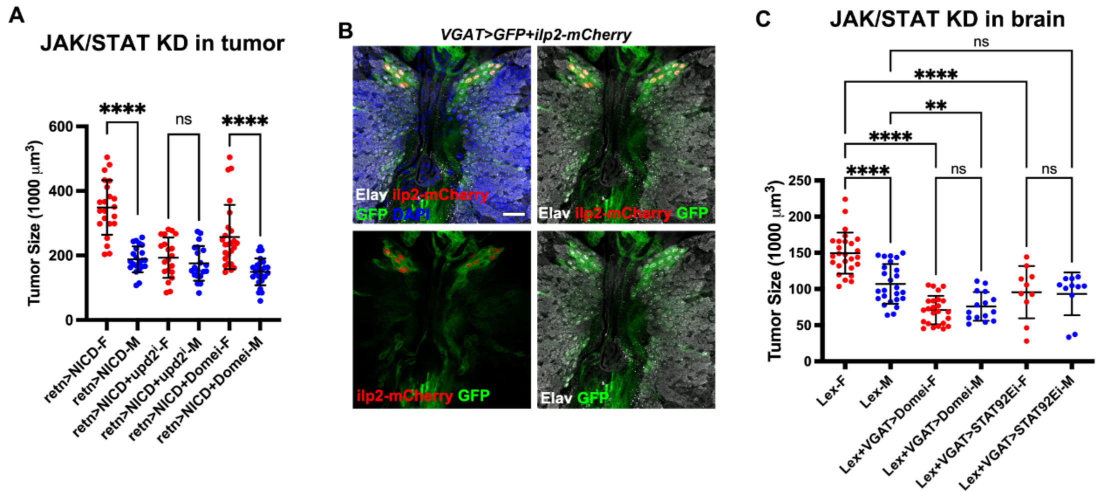

Fig. S4. Systemic regulation of tumor growth by Upd2. (A) Tumor size analysis from *upd2* or *Dome* knockdown in *retn>NICD* tumors. (B) Confocal images showing the expression pattern of *VGAT-Gal4*. Larval brain from *VGAT>GFP+ilp2-mcherry* was stained with anti-Elav (gray) and DAPI (blue). Scale bars, 20 µm. (C) Tumor size analysis in larvae with reduced JAK/STAT activity in the brain. Data represent mean ± SD, with statistical analysis conducted using one-way ANOVA with Tukey's multiple comparisons test. ns, $p > 0.05$, **$p<0.01$, ****$p < 0.0001$.

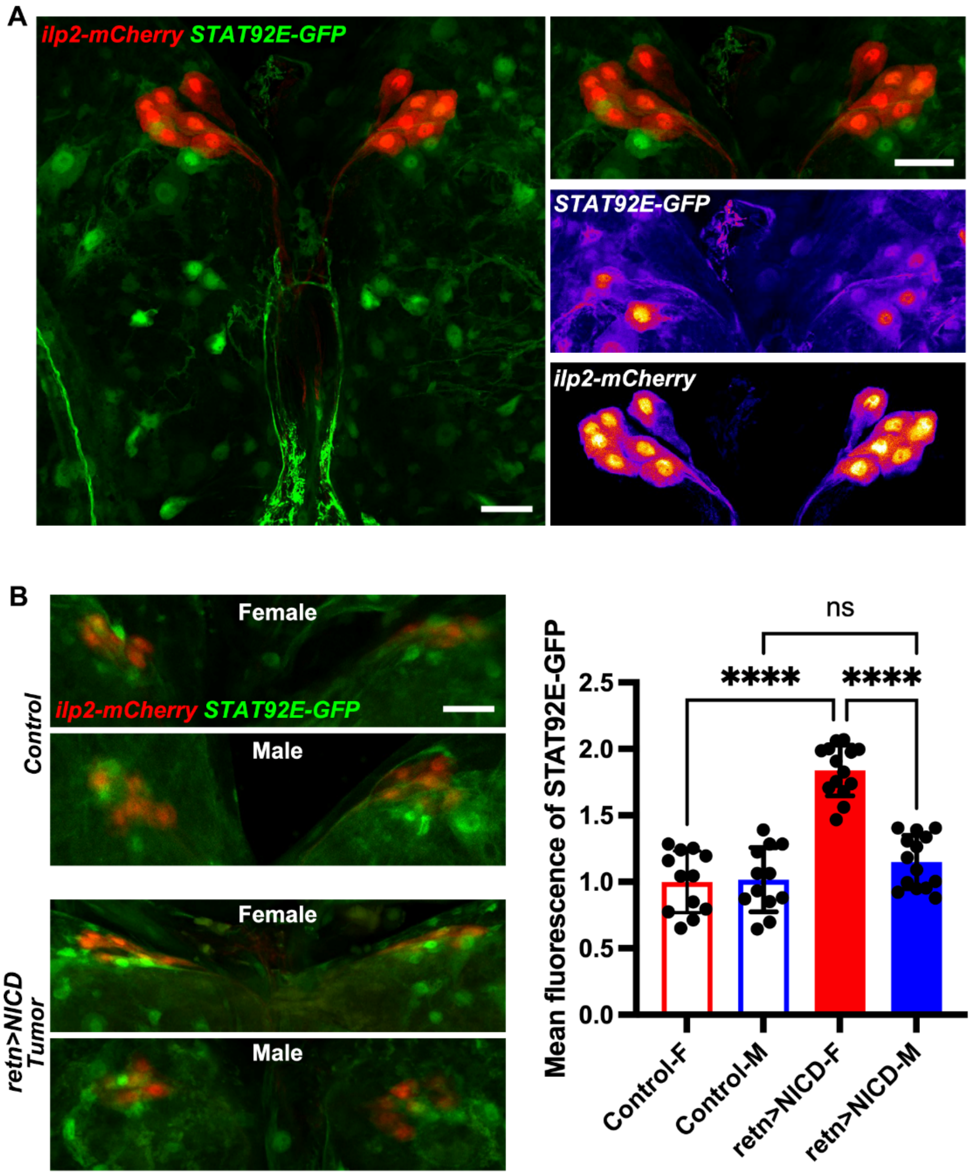

Fig. S5. JAK/STAT signaling controls the release of Dilp2 from IPCs. (A) A larval brain expressing ilp2-mCherry and STAT92E-GFP. Cell bodies of neurons expressing the STAT92E-GFP reporter are located next to the IPCs. The colors for STAT92E-GFP and ilp2-mcherry (right panel) were generated using 'LUT-Fire' plugin in ImageJ. (B) The expression of STAT92E-GFP in the control or tumor-bearing larval brains. Graph showing the quantification of STAT92E-GFP intensity. GFP intensity in GABA neurons adjacent to insulin-producing cells was measured. The GFP intensity of the female control was set as a baseline of one, with intensities from other samples adjusted relative to this control. Scale bars: 20 µm. Data represent mean ± SD, with statistical analysis conducted using one-way ANOVA with Tukey's multiple comparisons test. ns, $p > 0.05$; ****$p < 0.0001$.

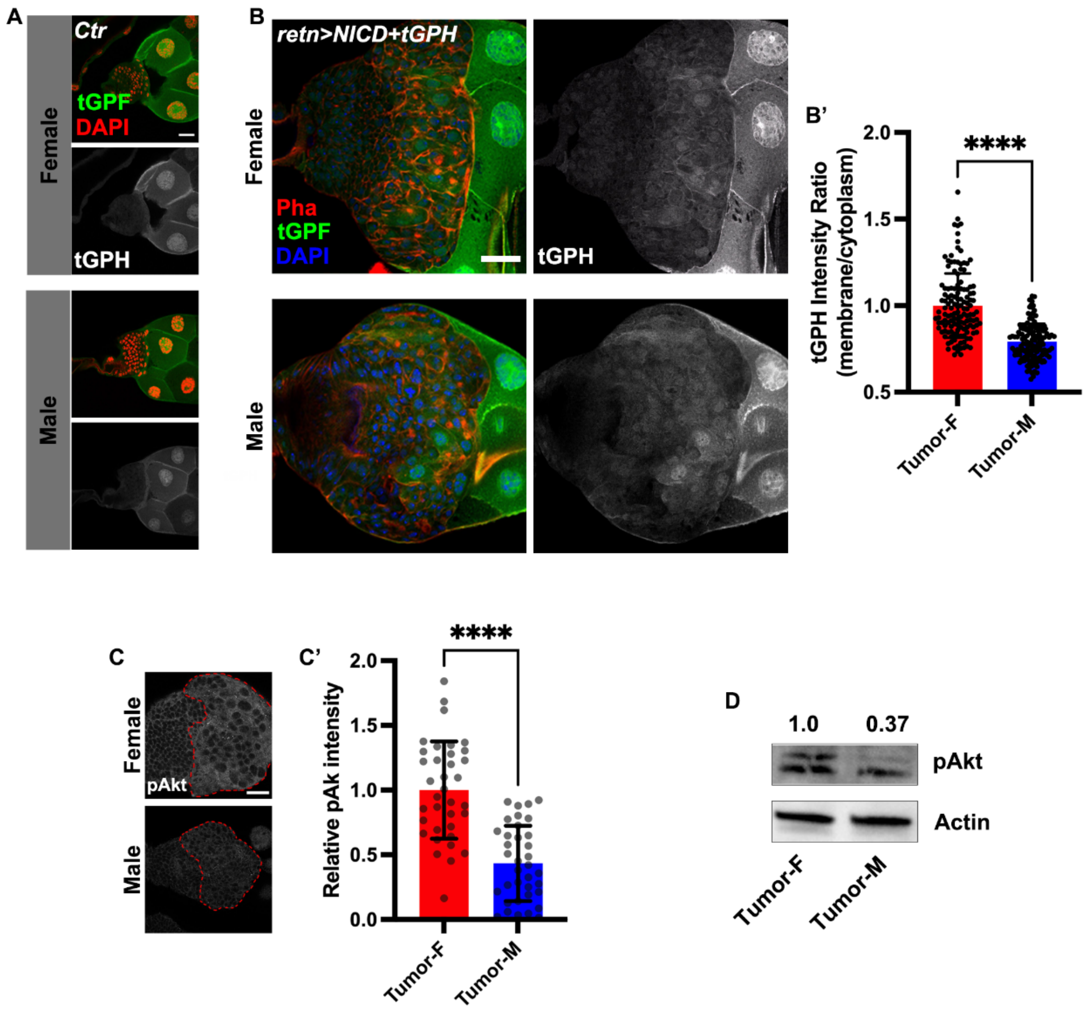

Fig. S6. Analysis of insulin levels in control and tumor Imaginal Rings (ImRs). (A) tGPH expression in control female and male larval ImRs. (B, B') ImR tumors with the IIS reporter (tGPH) were stained with Phalloidin (red) and DAPI (blue). A graph shows the intensity ratio of GFP between the cell membrane and the cytoplasm. (C) Female and male NICD-TZ tumors (outlined by red-dashed lines) were stained with the pAkt antibody. (C') Graph shows the fluorescence intensity of pAkt staining between female and male tumor regions. Mean fluorescence of pAkt was measured from the anterior non-tumor region and the posterior tumor region from each individual confocal image. The intensity from the non-tumor region was subtracted from that of the tumor region to determine the pAkt intensity for each sample. The 'relative pAkt intensity' refers to the comparison with the pAkt intensity from female tumors. (D) Western blot analysis of pAkt in female and male NICD-TZ tumors. Numbers indicate relative pAkt levels normalized to Actin levels. Mean values and SD are shown in graphs. Student's t test, ****$p<0.0001$. Scale bars: 20 μm.

Table S1. Primers for RT-qPCR

Primers	*Reference*
GAPDH-F: TAAATTCGACTCGACTCACGGT	*(99)*
GAPDH-R: CTCCACCACATACTCGGCTC	*(99)*
Mmp1-F: TCAGTGTTCATAGTCGTAGGCA	*(100)*
Mmp1-R: GGTAGATAGCCGAACTGGGAC	*(100)*
egr-F: CAGTGCATCCTCAGCCTCAA	This paper
egr-R: GCAGGGCTCTCTTTGGAAGT	This paper
upd2-F: CGGAACATCACGATGAGCGAAT	*(64)*
upd2-R: TCGGCAGGAACTTGTACTCG	*(64)*
Dilp2-F: GTATGGTGTGCGAGGAGTAT	*(64)*
Dilp2-R: TGAGTACACCCCCAAGATAG	*(64)*
bmm-F: GTCCCTTCAGTCCCTCCTTC	*(66)*
bmm-R: TATGAAGCACGCACACAACA	*(66)*
4E-BP-F: GCTAAGATGTCCGCTTCACC	*(66)*
4E-BP-R: CCTCCAGGAGTGGTGGAGTA	*(66)*

Table S2. Fly stocks and detailed genotypes

Fly stocks	***Source***
Act-Gal4/CyO	BDSC#4414
Mmp1-Gal4	BDSC#76161
retn-Gal4	BDSC#47433
Hml-Gal4,UAS-GFP	BDSC#30142
R4-Gal4	BDSC#33832
retn-lexA	BDSC#52608
Act-Gal4,UAS-mCD8GFP/CyO; Gal80ts	*(36)*
Mmp1-Gal4,UAS-mCD8GFP/CyO; Gal80ts	*(36)*
retn-Gal4,UAS-mCD8GFP	This study
VGTA-Gal4	BDSC#58409
Lpp-Gal4	BDSC#84317
TRE-RFP	*(39)*
ilp2-mCherry	BDSC#80707
STAT92E-GFP	BDSC#26198
Diap1-GFP	*(101)*
Fly-FUCCI	BDSC#55124
tGPH	BDSC#8164
UAS-NICD	*(35)*
lexAop-NICD	Wu-Min Deng lab
UAS-puc	*(102)*
UAS-mCD8RFP	BDSC#32220
UAS-Tak1RNAi	BDSC#33404
UAS-hepRNAi	BDSC#35210
UAS-bskRNAi	BDSC#53310
UAS-pucRNAi	BDSC#57300
UAS-GrndRNAi	VDRC#104538
UAS-egrRNAi	VDRC#45252
UAS-traRNAi	BDSC#44109
UAS-SxlRNAi	BDSC#34393
UAS-upd2RNAi	BDSC#33988
UAS-InRRNAi	BDSC#31307
UAS-Pi3KRNAi	BDSC#27690
UAS-InRDN	*BDSC*#8253
UAS-DomeDN	*(62)*

upd2Delta	BDSC#55727

Detailed Genotypes

Figure	***Panel***	***Genotype***
**Figure 1**	B, C	yw; UAS-NICD/+; retn-Gal4,UAS-mCD8GFP/+
	D-Female	UAS-mCD8RFP/yw; Act-Gal4,UAS-mCD8GFP/UAS-NICD; Gal80ts/+
	D-Male	yw/Y; Act-Gal4,UAS-mCD8GFP/UAS-NICD; Gal80ts/+
	E	yw; Mmp1-Gal4, UAS-mCD8GFP/UAS-NICD; Gal80ts/+
	F	yw; retn-LexA/+; LexAop-NICD/+
	G, G', G"	yw; Act-Gal4,UAS-mCD8GFP/+; Gal80ts/+
**Figure 2**	A left panel	TRE-RFP
	A right panel	yw; Act-Gal4,UAS-mCD8GFP/UAS-NICD; Gal80ts/TRE-RFP
	B Ctr	yw; Act-Gal4,UAS-mCD8GFP/+; Gal80ts/+
	B Tumor	yw; Act-Gal4,UAS-mCD8GFP/UAS-NICD; Gal80ts/+
	C NICD	yw; Act-Gal4,UAS-mCD8GFP/UAS-NICD; Gal80ts/+
	C NICD+bski	yw; Act-Gal4,UAS-mCD8GFP/UAS-NICD; Gal80ts/UAS-bski
	C NICD+Tak1i	yw; Act-Gal4,UAS-mCD8GFP/UAS-NICD; Gal80ts/UAS-Tak1RNAi
	C NICD+hepi	yw; Act-Gal4,UAS-mCD8GFP/UAS-NICD; Gal80ts/UAS-hepRNAi
**Figure 3**	A left panel	yw; Mmp1-Gal4/+; +/+
	A right panel	yw; Mmp1-Gal4, UAS-mCD8GFP/UAS-NICD; +/+
	B	yw; Mmp1-Gal4, UAS-mCD8GFP/UAS-NICD; +/+
	C NICD	yw; Act-Gal4,UAS-mCD8GFP/UAS-NICD; Gal80ts/+
	C NICD+GrndRNAi	yw; Act-Gal4,UAS-mCD8GFP/UAS-NICD; Gal80ts/UAS-GrndRNAi
	C NICD+egrRNAi	yw; Act-Gal4,UAS-mCD8GFP/UAS-NICD; Gal80ts/UAS-egrRNAi
	D, E	yw; Act-Gal4,UAS-mCD8GFP/UAS-NICD; Gal80ts/+
	F control	yw; UAS-NICD; +/+
	F tumor I	yw; Act-Gal4,UAS-mCD8GFP/UAS-NICD; Gal80ts/+
	F Tumor II	yw; Mmp1-Gal4, UAS-mCD8GFP/UAS-NICD; +/+
	G Lex	yw; retn-LexA/+; LexAop-NICD/+
	G Hml>EgrRNAi	yw; retn-LexA/UAS-EgrRNAi; LexAop-NICD/Hml>GFP
	G R4>EgrRNAi	yw; retn-LexA/UAS-EgrRNAi; LexAop-NICD/R4-Gal4
	G Lpp>EgrRNAi	yw; retn-LexA/UAS-EgrRNAi; LexAop-NICD/Lpp-Gal4
	H Lex	yw; retn-LexA/+; LexAop-NICD/+
	H Hml>traRNAi	yw; retn-LexA/UAS-traRNAi; LexAop-NICD/Hml>GFP
	H Hml>tra	yw; retn-LexA/UAS-tra; LexAop-NICD/Hml>GFP
	I Lex	yw; retn-LexA/+; LexAop-NICD/+
	I Hml>traRNAi	yw; retn-LexA/UAS-traRNAi; LexAop-NICD/Hml>GFP
	I Hml>SxlRNAi	yw; retn-LexA/LexAop-NICD; UAS-SxlRNAi/Hml-Gal4
	I Hml>tra	yw; retn-LexA/UAS-tra; LexAop-NICD/Hml>GFP

**Figure 4**	A Control	yw; Act-Gal4,UAS-mCD8GFP/+; Gal80ts/+
	A Tumor	yw; Act-Gal4,UAS-mCD8GFP/UAS-NICD; Gal80ts/+
	B Lex	yw; retn-LexA/+; LexAop-NICD/+
	B Hml>traRNAi	yw; retn-LexA/UAS-traRNAi; LexAop-NICD/Hml>GFP
	B Hml>tra	yw; retn-LexA/UAS-tra; LexAop-NICD/Hml>GFP
	C (i), D (i)	yw; Act-Gal4,UAS-mCD8GFP/UAS-NICD; Gal80ts/+
	C (ii), D (ii)	yw; Act-Gal4,UAS-mCD8GFP/UAS-NICD; Gal80ts/Tak1RNAi
	C (iii), D (iii)	yw; Act-Gal4,UAS-mCD8GFP/UAS-NICD; Gal80ts/bskRNAi
	C (iv), D (iv)	yw; Act-Gal4,UAS-mCD8GFP/UAS-NICD,UAS-pucRNAi; Gal80ts/+
	E NICD	yw; UAS-NICD/+; retn-Gal4,UAS-mCD8GFP/+
	E NICD+DomeDN	yw; UAS-NICD/+; retn-Gal4,UAS-mCD8GFP/UAS-DomeDN
	E upd2Delta; NICD	upd2Delta;UAS-NICD/+; retn-Gal4,UASmCD8GFP/+
**Figure 5**	B, C, D Control	yw; +/+; retn-Gal4,UAS-mCD8GFP/+
	B, C, D Tumor	yw; UAS-NICD/+; retn-Gal4,UAS-mCD8GFP/+
	D	yw; Mmp1-Gal4,UAS-mCD8GFP/UAS-NICD; Gal80ts/+
	E, F NICD	yw; Act-Gal4,UAS-mCD8GFP/UAS-NICD; Gal80ts/+
	E, F NICD+upd2RNAi	yw; Act-Gal4,UAS-mCD8GFP/UAS-NICD; Gal80ts/UAS-upd2RNAi
**Figure 6**	B, C Control	yw; Act-Gal4,UAS-mCD8GFP/+; Gal80ts/+
	B, C Tumor	yw; Act-Gal4,UAS-mCD8GFP/UAS-NICD; Gal80ts/+
	E Lex	yw; retn-LexA/+; LexAop-NICD/+
	E Hml>traRNAi	yw; retn-LexA/UAS-traRNAi; LexAop-NICD/Hml>GFP
	E Hml>tra	yw; retn-LexA/UAS-tra; LexAop-NICD/Hml>GFP
	F NICD	yw; UAS-NICD/+; retn-Gal4,UAS-mCD8GFP/+
	F NICD+InRRNAi	yw; UAS-NICD/+; retn-Gal4,UAS-mCD8GFP/UAS-InRRNAi
	F NICD+InRDN	yw; UAS-NICD/+; retn-Gal4,UAS-mCD8GFP/UAS-InRDN
	F NICD+Pi3KRNAi	yw; UAS-NICD/+; retn-Gal4,UAS-mCD8GFP/UAS-Pi3KRNAi
Figure S1	Actts>mCD8GFP	yw; Act-Gal4,UAS-mCD8GFP/+; Gal80ts/+
	Actts>mCD8GFP+NICD	yw; Act-Gal4,UAS-mCD8GFP/UAS-NICD; Gal80ts/+
	Mpts>mCD8GFP	yw; Mmp1-Gal4,UAS-mCD8GFP/+; Gal80ts/+
	Mpts>mCD8GFP+NICD	yw; Mmp1-Gal4,UAS-mCD8GFP/UAS-NICD; Gal80ts/+
	retn>mCD8GFP	yw; +/+; retn-Gal4,UAS-mCD8GFP/+
	retn>mCD8GFP+NICD	yw; UAS-NICD/+; retn-Gal4,UAS-mCD8GFP/+
Figure S2	A, A’, A’’	yw; Mmp1-Gal4,UAS-mCD8GFP/UAS-NICD; Gal80ts/+
	B left panel	yw; +/+; Diap1-GFP
	B right panel	yw; Act-Gal4/UAS-NICD; Gal80ts/Diap1-GFP
	C	yw; Act-Gal4/UAS-NICD; Gal80ts/ubi-E2f1-GFP, ubi-CycB-RFP.nls
	D, E, F	yw; Act-Gal4,UAS-mCD8GFP/UAS-NICD; Gal80ts/+

Figure S3	A retn>NICD	yw; UAS-NICD/+; retn-Gal4,UAS-mCD8GFP/+
	A egr1/egr1; retn>NICD	yw; egr1/egr1; retn-Gal4,UAS-mCD8GFP/UAS-NICD
	B retn>GFP	yw; +/+; retn-Gal4,UAS-mCD8GFP/+
	B retn>NICD	yw; UAS-NICD/+; retn-Gal4,UAS-mCD8GFP/+
	B Mmp1>NICD	yw; Mmp1-Gal4,UAS-mCD8GFP/UAS-NICD; +/+
	C	yw; Mmp1-Gal4,UAS-mCD8GFP/UAS-NICD; +/+
	D	yw; retn-LexA/+; LexAop-NICD/Hml>GFP
	E Ctr	yw; +/+; retn-Gal4,UAS-mCD8GFP/+
	E Tumor	yw; UAS-NICD/+; retn-Gal4,UAS-mCD8GFP/+
	F retn>NICD	yw; UAS-NICD/+; retn-Gal4,UAS-mCD8GFP/+
	F retn>NICD+trai	yw; UAS-NICD/+; retn-Gal4,UAS-mCD8GFP/UAS-traRNAi
	F retn>NICD+tra	yw; UAS-NICD/+; retn-Gal4,UAS-mCD8GFP/UAS-tra
Figure S4	A retn>NICD	yw; UAS-NICD/+; retn-Gal4,UAS-mCD8GFP/+
	A retn>NICD+upd2i	yw; UAS-NICD/+; retn-Gal4,UAS-mCD8GFP/UAS-upd2RNAi
	A retn>NICD+Domei	yw; UAS-NICD/+; retn-Gal4,UAS-mCD8GFP/UAS-DomeRNAi
	B VGAT>GFP+ilp2-mCherry	yw; UAS-mCD8GFP/+; VGAT-Gal4/ilp2-mCherry
	C Lex	yw; retn-LexA/+; LexAop-NICD/+
	C Lex+VGAT>Domei	yw; retn-LexA/LexAop-NICD; VGAT-Gal4/UAS-DomeRNAi
	C Lex+VGAT>STAT92Ei	yw; retn-LexA/LexAop-NICD; VGAT-Gal4/UAS-STAT92ERNAi
Figure S5	A	yw; 10XSTAT92E-GFP; ilp2-mCherry
	B Control	yw; 10XSTAT92E-GFP/+; ilp2-mCherry/retn-Gal4
	B retn>NICD Tumor	yw; 10XSTAT92E-GFP/UAS-NICD; ilp2-mCherry/retn-Gal4
Figure S6	A	tGPH
	B	yw; UAS-NICD/+; retn-Gal4/tGPH
	C	yw; Act-Gal4,UAS-mCD8GFP/UAS-NICD; Gal80ts/+
	D	yw; UAS-NICD/+; retn-Gal4,UAS-mCD8GFP/+

REFERENCES AND NOTES

1. D. Hanahan, R. A. Weinberg, Hallmarks of cancer: The next generation. *Cell* **144**, 646–674 (2011).

2. C. R. Johnson, H. D. Thames, D. T. Huang, R. K. Schmidt-Ullrich, The tumor volume and clonogen number relationship: Tumor control predictions based upon tumor volume estimates derived from computed tomography. *Int. J. Radiat. Oncol. Biol. Phys.* **33**, 281–287 (1995).

3. M. K. Chen, T. H. Chen, J. P. Liu, C. C. Chang, W. C. Chie, Better prediction of prognosis for patients with nasopharyngeal carcinoma using primary tumor volume. *Cancer* **100**, 2160–2166 (2004).

4. P. Goldstraw, J. Crowley, K. Chansky, D. J. Giroux, P. A. Groome, R. Rami-Porta, P. E. Postmus, V. Rusch, L. Sobin; International Association for the Study of Lung Cancer International Staging, Committee and Participating Institutions, The IASLC Lung Cancer Staging Project: Proposals for the revision of the TNM stage groupings in the forthcoming (seventh) edition of the TNM Classification of malignant tumours. *J. Thorac. Oncol.* **2**, 706–714 (2007).

5. D. F. Quail, J. A. Joyce, Microenvironmental regulation of tumor progression and metastasis. *Nat. Med.* **19**, 1423–1437 (2013).

6. A. Labani-Motlagh, M. Ashja-Mahdavi, A. Loskog, The tumor microenvironment: A milieu hindering and obstructing antitumor immune responses. *Front Immunol.* **11**, 940 (2020).

7. X. Mao, J. Xu, W. Wang, C. Liang, J. Hua, J. Liu, B. Zhang, Q. Meng, X. Yu, S. Shi, Crosstalk between cancer-associated fibroblasts and immune cells in the tumor microenvironment: New findings and future perspectives. *Mol. Cancer* **20**, 131 (2021).

8. N. M. Anderson, M. C. Simon, The tumor microenvironment. *Curr. Biol.* **30**, R921–R925 (2020).

9. S. S. McAllister, R. A. Weinberg, The tumour-induced systemic environment as a critical regulator of cancer progression and metastasis. *Nat. Cell Biol.* **16**, 717–727 (2014).

10. E. J. Gallagher, D. LeRoith, Minireview: IGF, insulin, and cancer. *Endocrinology* **152**, 2546–2551 (2011).

11. D. B. Ulanet, D. L. Ludwig, C. R. Kahn, D. Hanahan, Insulin receptor functionally enhances multistage tumor progression and conveys intrinsic resistance to IGF-1R targeted therapy. *Proc. Natl. Acad. Sci. U.S.A.* **107**, 10791–10798 (2010).

12. R. Novosyadlyy, D. E. Lann, A. Vijayakumar, A. Rowzee, D. A. Lazzarino, Y. Fierz, J. M. Carboni, M. M. Gottardis, P. A. Pennisi, A. A. Molinolo, N. Kurshan, W. Mejia, S. Santopietro, S. Yakar, T. L. Wood, D. LeRoith, Insulin-mediated acceleration of breast cancer development and progression in a nonobese model of type 2 diabetes. *Cancer Res.* **70**, 741–751 (2010).

13. Y. Fierz, R. Novosyadlyy, A. Vijayakumar, S. Yakar, D. LeRoith, Insulin-sensitizing therapy attenuates type 2 diabetes-mediated mammary tumor progression. *Diabetes* **59**, 686–693 (2010).

14. K. Masur, C. Vetter, A. Hinz, N. Tomas, H. Henrich, B. Niggemann, K. S. Zanker, Diabetogenic glucose and insulin concentrations modulate transcriptome and protein levels involved in tumour cell migration, adhesion and proliferation. *Br. J. Cancer* **104**, 345–352 (2011).

15. I. F. Godsland, Insulin resistance and hyperinsulinaemia in the development and progression of cancer. *Clin. Sci.* **118**, 315–332 (2010).

16. B. Arcidiacono, S. Iiritano, A. Nocera, K. Possidente, M. T. Nevolo, V. Ventura, D. Foti, E. Chiefari, A. Brunetti, Insulin resistance and cancer risk: An overview of the pathogenetic mechanisms. *Exp. Diabetes Res.* **2012**, 789174 (2012).

17. S. Tsugane, M. Inoue, Insulin resistance and cancer: Epidemiological evidence. *Cancer Sci.* **101**, 1073–1079 (2010).

18. H. I. Kim, H. Lim, A. Moon, Sex differences in cancer: Epidemiology, genetics and therapy. *Biomol. Ther.* **26**, 335–342 (2018).

19. R. L. Siegel, K. D. Miller, H. E. Fuchs, A. Jemal, Cancer statistics, 2022. *CA Cancer J. Clin.* **72**, 7–33 (2022).

20. J. B. Rubin, J. S. Lagas, L. Broestl, J. Sponagel, N. Rockwell, G. Rhee, S. F. Rosen, S. Chen, R. S. Klein, P. Imoukhuede, J. Luo, Sex differences in cancer mechanisms. *Biol. Sex Differ.* **11**, 17 (2020).

21. I. Lucca, T. Klatte, H. Fajkovic, M. de Martino, S. F. Shariat, Gender differences in incidence and outcomes of urothelial and kidney cancer. *Nat. Rev. Urol.* **12**, 585–592 (2015).

22. S. H. Giordano, D. S. Cohen, A. U. Buzdar, G. Perkins, G. N. Hortobagyi, Breast carcinoma in men: A population-based study. *Cancer* **101**, 51–57 (2004).

23. L. Gabriele, M. Buoncervello, B. Ascione, M. Bellenghi, P. Matarrese, A. Care, The gender perspective in cancer research and therapy: Novel insights and on-going hypotheses. *Ann. Ist. Super. Sanita* **52**, 213–222 (2016).

24. M. T. Dorak, E. Karpuzoglu, Gender differences in cancer susceptibility: An inadequately addressed issue. *Front. Genet.* **3**, 268 (2012).

25. S. L. Klein, K. L. Flanagan, Sex differences in immune responses. *Nat. Rev. Immunol.* **16**, 626–638 (2016).

26. P. D. DiMusto, G. Lu, A. Ghosh, K. J. Roelofs, O. Sadiq, B. McEvoy, G. Su, A. Laser, C. M. Bhamidipati, G. Ailawadi, P. K. Henke, J. L. Eliason, G. R. Upchurch Jr., Increased JNK in males compared with females in a rodent model of abdominal aortic aneurysm. *J. Surg. Res.* **176**, 687–695 (2012).

27. S. Win, T. A. Than, N. Kaplowitz, The regulation of JNK signaling pathways in cell death through the interplay with mitochondrial SAB and upstream post-translational effects. *Int. J. Mol. Sci.* **19**, 3657 (2018).

28. C. L. White, W. S. N. Jayasekara, D. Picard, J. Chen, D. N. Watkins, J. E. Cain, M. Remke, D. J. Gough, A sexually dimorphic role for STAT3 in sonic hedgehog medulloblastoma. *Cancers* **11**, 1702 (2019).

29. C. Molnar, J. P. Heinen, J. Reina, S. Llamazares, E. Palumbo, A. Breschi, M. Gay, L. Villarreal, M. Vilaseca, G. Pollarolo, C. Gonzalez, The histone code reader PHD finger protein 7 controls sex-linked disparities in gene expression and malignancy in *Drosophila*. *Sci. Adv.* **5**, eaaw7965 (2019).

30. H. Herranz, S. M. Cohen, *Drosophila* as a model to study the link between metabolism and cancer. *J. Dev. Biol.* **5**, 15 (2017).

31. D. Bilder, K. Ong, T. C. Hsi, K. Adiga, J. Kim, Tumour-host interactions through the lens of *Drosophila*. *Nat. Rev. Cancer* **21**, 687–700 (2021).

32. Y. Liu, P. Saavedra, N. Perrimon, Cancer cachexia: Lessons from *Drosophila*. *Dis. Model Mech.* **15**, dmm049298 (2022).

33. E. Bier, *Drosophila*, the golden bug, emerges as a tool for human genetics. *Nat. Rev. Genet.* **6**, 9–23 (2005).

34. B. Ugur, K. Chen, H. J. Bellen, *Drosophila* tools and assays for the study of human diseases. *Dis. Model Mech.* **9**, 235–244 (2016).

35. S. A. Yang, J. M. Portilla, S. Mihailovic, Y. C. Huang, W. M. Deng, Oncogenic notch triggers neoplastic tumorigenesis in a transition-zone-like tissue microenvironment. *Dev. Cell* **49**, 461–472.e5 (2019).

36. X. F. Wang, S. A. Yang, S. Gong, C. H. Chang, J. M. Portilla, D. Chatterjee, J. Irianto, H. Bao, Y. C. Huang, W. M. Deng, Polyploid mitosis and depolyploidization promote chromosomal instability and tumor progression in a Notch-induced tumor model. *Dev. Cell* **56**, 1976–1988.e4 (2021).

37. M. Lecourtois, F. Schweisguth, Indirect evidence for *Delta*-dependent intracellular processing of notch in *Drosophila* embryos. *Curr. Biol.* **8**, 771–775 (1998).

38. N. Zielke, J. Korzelius, M. van Straaten, K. Bender, G. F. P. Schuhknecht, D. Dutta, J. Xiang, B. A. Edgar, Fly-FUCCI: A versatile tool for studying cell proliferation in complex tissues. *Cell Rep.* **7**, 588–598 (2014).

39. N. Chatterjee, D. Bohmann, A versatile ΦC31 based reporter system for measuring AP-1 and Nrf2 signaling in *Drosophila* and in tissue culture. *PLOS ONE* **7**, e34063 (2012).

40. A. Page-McCaw, J. Serano, J. M. Sante, G. M. Rubin, *Drosophila* matrix metalloproteinases are required for tissue remodeling, but not embryonic development. *Dev. Cell* **4**, 95–106 (2003).

41. A. Ozturk-Colak, S. J. Marygold, G. Antonazzo, H. Attrill, D. Goutte-Gattat, V. K. Jenkins, B. B. Matthews, G. Millburn, G. Dos Santos, C. J. Tabone, C. FlyBase, FlyBase: Updates to the *Drosophila* genes and genomes database. *Genetics* **227**, iyad211 (2024).

42. J. E. La Marca, H. E. Richardson, Two-faced: Roles of JNK signalling during tumourigenesis in the *Drosophila* model. *Front. Cell Dev. Biol.* **8**, 42 (2020).

43. D. S. Andersen, J. Colombani, V. Palmerini, K. Chakrabandhu, E. Boone, M. Rothlisberger, J. Toggweiler, K. Basler, M. Mapelli, A. O. Hueber, P. Leopold, The *Drosophila* TNF receptor grindelwald couples loss of cell polarity and neoplastic growth. *Nature* **522**, 482–486 (2015).

44. H. Kanda, T. Igaki, H. Kanuka, T. Yagi, M. Miura, Wengen, a member of the *Drosophila* tumor necrosis factor receptor superfamily, is required for Eiger signaling. *J. Biol. Chem.* **277**, 28372–28375 (2002).

45. T. Igaki, H. Kanda, Y. Yamamoto-Goto, H. Kanuka, E. Kuranaga, T. Aigaki, M. Miura, Eiger, a TNF superfamily ligand that triggers the *Drosophila* JNK pathway. *EMBO J.* **21**, 3009–3018 (2002).

46. E. Moreno, M. Yan, K. Basler, Evolution of TNF signaling mechanisms: JNK-dependent apoptosis triggered by Eiger, the *Drosophila* homolog of the TNF superfamily. *Curr. Biol.* **12**, 1263–1268 (2002).

47. M. A. Bhat, S. Izaddoost, Y. Lu, K. O. Cho, K. W. Choi, H. J. Bellen, Discs Lost, a novel multi-PDZ domain protein, establishes and maintains epithelial polarity. *Cell* **96**, 833–845 (1999).

48. W. Zhou, Y. Hong, *Drosophila* Patj plays a supporting role in apical-basal polarity but is essential for viability. *Development* **139**, 2891–2896 (2012).

49. F. Parisi, R. K. Stefanatos, K. Strathdee, Y. Yu, M. Vidal, Transformed epithelia trigger non-tissue-autonomous tumor suppressor response by adipocytes via activation of Toll and Eiger/TNF signaling. *Cell Rep.* **6**, 855–867 (2014).

50. N. Agrawal, R. Delanoue, A. Mauri, D. Basco, M. Pasco, B. Thorens, P. Leopold, The *Drosophila* TNF Eiger is an adipokine that acts on insulin-producing cells to mediate nutrient response. *Cell Metab.* **23**, 675–684 (2016).

51. M. Muzzopappa, L. Murcia, M. Milan, Feedback amplification loop drives malignant growth in epithelial tissues. *Proc. Natl. Acad. Sci. U.S.A.* **114**, E7291–E7300 (2017).

52. C. E. Fogarty, N. Diwanji, J. L. Lindblad, M. Tare, A. Amcheslavsky, K. Makhijani, K. Bruckner, Y. Fan, A. Bergmann, Extracellular reactive oxygen species drive apoptosis-induced proliferation via *Drosophila* macrophages. *Curr. Biol.* **26**, 575–584 (2016).

53. G. de Vreede, S. U. Gerlach, D. Bilder, Epithelial monitoring through ligand-receptor segregation ensures malignant cell elimination. *Science* **376**, 297–301 (2022).

54. B. S. Baker, Sex in flies: The splice of life. *Nature* **340**, 521–524 (1989).

55. A. Sawala, A. P. Gould, The sex of specific neurons controls female body growth in *Drosophila*. *PLOS Biol.* **15**, e2002252 (2017).

56. J. C. Pastor-Pareja, M. Wu, T. Xu, An innate immune response of blood cells to tumors and tissue damage in Drosophila. *Dis. Model. Mech.* **1**, 144–154 (2008).

57. M. Wu, J. C. Pastor-Pareja, T. Xu, Corrigendum: Interaction between Ras^{V12} and scribbled clones induces tumour growth and invasion. *Nature* **543**, 452 (2017).

58. H. Jiang, P. H. Patel, A. Kohlmaier, M. O. Grenley, D. G. McEwen, B. A. Edgar, Cytokine/Jak/Stat signaling mediates regeneration and homeostasis in the *Drosophila* midgut. *Cell* **137**, 1343–1355 (2009).

59. B. K. Staley, K. D. Irvine, Warts and Yorkie mediate intestinal regeneration by influencing stem cell proliferation. *Curr. Biol.* **20**, 1580–1587 (2010).

60. J. C. Hombria, S. Brown, S. Hader, M. P. Zeidler, Characterisation of Upd2, a *Drosophila* JAK/STAT pathway ligand. *Dev. Biol.* **288**, 420–433 (2005).

61. M. P. Zeidler, E. A. Bach, N. Perrimon, The roles of the *Drosophila* JAK/STAT pathway. *Oncogene* **19**, 2598–2606 (2000).

62. S. Brown, N. Hu, J. C. Hombria, Identification of the first invertebrate interleukin JAK/STAT receptor, the *Drosophila* gene *domeless*. *Curr. Biol.* **11**, 1700–1705 (2001).

63. D. Romao, M. Muzzopappa, L. Barrio, M. Milan, The Upd3 cytokine couples inflammation to maturation defects in *Drosophila*. *Curr. Biol.* **31**, 1780–1787.e6 (2021).

64. A. Rajan, N. Perrimon, *Drosophila* cytokine unpaired 2 regulates physiological homeostasis by remotely controlling insulin secretion. *Cell* **151**, 123–137 (2012).

65. H. Fei, D. M. Chow, A. Chen, R. Romero-Calderon, W. S. Ong, L. C. Ackerson, N. T. Maidment, J. H. Simpson, M. A. Frye, D. E. Krantz, Mutation of the *Drosophila* vesicular GABA transporter disrupts visual figure detection. *J. Exp. Biol.* **213**, 1717–1730 (2010).

66. J. W. Millington, G. P. Brownrigg, C. Chao, Z. Sun, P. J. Basner-Collins, L. W. Wat, B. Hudry, I. Miguel-Aliaga, E. J. Rideout, Female-biased upregulation of insulin pathway activity mediates the sex difference in *Drosophila* body size plasticity. *elife* **10**, e58341 (2021).

67. K. Blaschke, K. T. Ebata, M. M. Karimi, J. A. Zepeda-Martinez, P. Goyal, S. Mahapatra, A. Tam, D. J. Laird, M. Hirst, A. Rao, M. C. Lorincz, M. Ramalho-Santos, Vitamin C induces Tet-dependent DNA demethylation and a blastocyst-like state in ES cells. *Nature* **500**, 222–226 (2013).

68. B. Hudry, E. de Goeij, A. Mineo, P. Gaspar, D. Hadjieconomou, C. Studd, J. B. Mokochinski, H. B. Kramer, P. Y. Placais, T. Preat, I. Miguel-Aliaga, Sex differences in intestinal carbohydrate metabolism promote food intake and sperm maturation. *Cell* **178**, 901–918.e16 (2019).

69. J. S. Britton, W. K. Lockwood, L. Li, S. M. Cohen, B. A. Edgar, Drosophila's insulin/PI3-kinase pathway coordinates cellular metabolism with nutritional conditions. *Dev. Cell* **2**, 239–249 (2002).

70. E. M. Mabery, D. S. Schneider, The *Drosophila* TNF ortholog eiger is required in the fat body for a robust immune response. *J. Innate Immun.* **2**, 371–378 (2010).

71. J. B. Cordero, J. P. Macagno, R. K. Stefanatos, K. E. Strathdee, R. L. Cagan, M. Vidal, Oncogenic Ras diverts a host TNF tumor suppressor activity into tumor promoter. *Dev. Cell* **18**, 999–1011 (2010).

72. S. Wu, C. M. Boyer, R. S. Whitaker, A. Berchuck, J. R. Wiener, J. B. Weinberg, R. C. Bast Jr., Tumor necrosis factor alpha as an autocrine and paracrine growth factor for ovarian cancer: Monokine induction of tumor cell proliferation and tumor necrosis factor alpha expression. *Cancer Res.* **53**, 1939–1944 (1993).

73. A. E. Brent, A. Rajan, Insulin and Leptin/Upd2 exert opposing influences on synapse number in fat-sensing neurons. *Cell Metab.* **32**, 786–800.e7 (2020).

74. M. C. Ingaramo, J. A. Sanchez, N. Perrimon, A. Dekanty, Fat body p53 regulates systemic insulin signaling and autophagy under nutrient stress via *Drosophila* Upd2 repression. *Cell Rep.* **33**, 108321 (2020).

75. H. Yang, J. Kronhamn, J. O. Ekstrom, G. G. Korkut, D. Hultmark, JAK/STAT signaling in *Drosophila* muscles controls the cellular immune response against parasitoid infection. *EMBO Rep.* **16**, 1664–1672 (2015).

76. X. Zhao, J. Karpac, Muscle directs diurnal energy homeostasis through a myokine-dependent hormone module in *Drosophila*. *Curr. Biol.* **27**, 1941–1955.e6 (2017).

77. K. Kierdorf, F. Hersperger, J. Sharrock, C. M. Vincent, P. Ustaoglu, J. Dou, A. Gyoergy, O. Gross, D. E. Siekhaus, M. S. Dionne, Muscle function and homeostasis require cytokine inhibition of AKT activity in *Drosophila*. *elife* **9**, e51595 (2020).

78. J. Kim, H. C. Chuang, N. K. Wolf, C. J. Nicolai, D. H. Raulet, K. Saijo, D. Bilder, Tumor-induced disruption of the blood-brain barrier promotes host death. *Dev. Cell* **56**, 2712–2721.e4 (2021).

79. X. T. Cai, H. Li, M. Borch Jensen, E. Maksoud, J. Borneo, Y. Liang, S. R. Quake, L. Luo, P. Haghighi, H. Jasper, Gut cytokines modulate olfaction through metabolic reprogramming of glia. *Nature* **596**, 97–102 (2021).

80. S. Chakrabarti, J. P. Dudzic, X. Li, E. J. Collas, J. P. Boquete, B. Lemaitre, Remote control of intestinal stem cell activity by haemocytes in *Drosophila*. *PLOS Genet.* **12**, e1006089 (2016).

81. X. Cao, M. Rojas, J. C. Pastor-Pareja, Intrinsic and damage-induced JAK/STAT signaling regulate developmental timing by the *Drosophila* prothoracic gland. *Dis. Model. Mech.* **15**, dmm049160 (2022).

82. Z. Zhai, S. Kondo, N. Ha, J. P. Boquete, M. Brunner, R. Ueda, B. Lemaitre, Accumulation of differentiating intestinal stem cell progenies drives tumorigenesis. *Nat. Commun.* **6**, 10219 (2015).

83. C. Garofalo, E. Surmacz, Leptin and cancer. *J. Cell Physiol.* **207**, 12–22 (2006).

84. C. Jimenez-Cortegana, A. Lopez-Saavedra, F. Sanchez-Jimenez, A. Perez-Perez, J. Castineiras, J. A. Virizuela-Echaburu, L. de la Cruz-Merino, V. Sanchez-Margalet, Leptin, both bad and good actor in cancer. *Biomolecules* **11**, 913 (2021).

85. M. H. Wu, Y. C. Chou, W. Y. Chou, G. C. Hsu, C. H. Chu, C. P. Yu, J. C. Yu, C. A. Sun, Circulating levels of leptin, adiposity and breast cancer risk. *Br. J. Cancer* **100**, 578–582 (2009).

86. S. Guo, M. Liu, G. Wang, M. Torroella-Kouri, R. R. Gonzalez-Perez, Oncogenic role and therapeutic target of leptin signaling in breast cancer and cancer stem cells. *Biochim. Biophys Acta.* **1825**, 207–222 (2012).

87. S. Chang, S. D. Hursting, J. H. Contois, S. S. Strom, Y. Yamamura, R. J. Babaian, P. Troncoso, P. S. Scardino, T. M. Wheeler, C. I. Amos, M. R. Spitz, Leptin and prostate cancer. *Prostate* **46**, 62–67 (2001).

88. R. L. Belmonte, M. K. Corbally, D. F. Duneau, J. C. Regan, Sexual dimorphisms in innate immunity and responses to infection in *Drosophila melanogaster*. *Front. Immunol.* **10**, 3075 (2020).

89. D. F. Duneau, H. C. Kondolf, J. H. Im, G. A. Ortiz, C. Chow, M. A. Fox, A. T. Eugenio, J. Revah, N. Buchon, B. P. Lazzaro, The Toll pathway underlies host sexual dimorphism in resistance to both Gram-negative and Gram-positive bacteria in mated *Drosophila*. *BMC Biol.* **15**, 124 (2017).

90. J. Schindelin, I. Arganda-Carreras, E. Frise, V. Kaynig, M. Longair, T. Pietzsch, S. Preibisch, C. Rueden, S. Saalfeld, B. Schmid, J. Y. Tinevez, D. J. White, V. Hartenstein, K. Eliceiri, P. Tomancak, A. Cardona, Fiji: An open-source platform for biological-image analysis. *Nat. Methods* **9**, 676–682 (2012).

91. X. F. Wang, J. X. Liu, Z. Y. Ma, Y. Shen, H. R. Zhang, Z. Z. Zhou, E. Suzuki, Q. X. Liu, S. Hirose, Evolutionarily conserved roles for apontic in induction and subsequent decline of cyclin e expression. *iScience* **23**, 101369 (2020).

92. Q. X. Liu, X. F. Wang, K. Ikeo, S. Hirose, W. J. Gehring, T. Gojobori, Evolutionarily conserved transcription factor Apontic controls the G1/S progression by inducing cyclin E during eye development. *Proc. Natl. Acad. Sci. U. S. A.* **111**, 9497–9502 (2014).

93. W. M. Deng, M. Schneider, R. Frock, C. Castillejo-Lopez, E. A. Gaman, S. Baumgartner, H. Ruohola-Baker, Dystroglycan is required for polarizing the epithelial cells and the oocyte in *Drosophila*. *Development* **130**, 173–184 (2003).

94. K. Nishioka, X. F. Wang, H. Miyazaki, H. Soejima, S. Hirose, Mbf1 ensures Polycomb silencing by protecting *E(z)* mRNA from degradation by Pacman. *Development* **145**, dev162461 (2018).

95. Q. Li, Z. Gong, Cold-sensing regulates *Drosophila* growth through insulin-producing cells. *Nat. Commun.* **6**, 10083 (2015).

96. A. Jevitt, D. Chatterjee, G. Xie, X. F. Wang, T. Otwell, Y. C. Huang, W. M. Deng, A single-cell atlas of adult *Drosophila* ovary identifies transcriptional programs and somatic cell lineage regulating oogenesis. *PLOS Biol.* **18**, e3000538 (2020).

97. D. Chatterjee, C. A. M. Costa, X. F. Wang, A. Jevitt, Y. C. Huang, W. M. Deng, Single-cell transcriptomics identifies Keap1-Nrf2 regulated collective invasion in a *Drosophila* tumor model. *elife* **11**, e80956 (2022).

98. Y. Hao, S. Hao, E. Andersen-Nissen, W. M. Mauck III, S. Zheng, A. Butler, M. J. Lee, A. J. Wilk, C. Darby, M. Zager, P. Hoffman, M. Stoeckius, E. Papalexi, E. P. Mimitou, J. Jain, A. Srivastava, T. Stuart, L. M. Fleming, B. Yeung, A. J. Rogers, J. M. McElrath, C. A. Blish, R. Gottardo, P. Smibert, R. Satija, Integrated analysis of multimodal single-cell data. *Cell* **184**, 3573–3587.e29 (2021).

99. F. Obata, C. O. Fons, A. P. Gould, Early-life exposure to low-dose oxidants can increase longevity via microbiome remodelling in *Drosophila*. *Nat. Commun.* **9**, 975 (2018).

100. W. Lodge, M. Zavortink, S. Golenkina, F. Froldi, C. Dark, S. Cheung, B. L. Parker, R. Blazev, D. Bakopoulos, E. L. Christie, V. C. Wimmer, B. C. Duckworth, H. E. Richardson, L. Y. Cheng, Tumor-derived MMPs regulate cachexia in a *Drosophila* cancer model. *Dev. Cell* **56**, 2664–2680.e6 (2021).

101. L. Zhang, F. Ren, Q. Zhang, Y. Chen, B. Wang, J. Jiang, The TEAD/TEF family of transcription factor Scalloped mediates Hippo signaling in organ size control. *Dev. Cell* **14**, 377–387 (2008).

102. E. Martin-Blanco, A. Gampel, J. Ring, K. Virdee, N. Kirov, A. M. Tolkovsky, A. Martinez-Arias, *Puckered* encodes a phosphatase that mediates a feedback loop regulating JNK activity during dorsal closure in *Drosophila*. *Genes Dev.* **12**, 557–570 (1998).
